# Chiropractic and CAM Utilization: A Descriptive Review

**DOI:** 10.1186/1746-1340-15-2

**Published:** 2007-01-22

**Authors:** Dana J Lawrence, William C Meeker

**Affiliations:** 1 Research Department, Palmer College of Chiropractic, 1000 Brady Street, Davenport, IA 52803 USA; 2 President, Palmer College of Chiropractic West, 90 E. Tasman Avenue, San Jose, CA 95134 USA

## Abstract

**Objective:**

To conduct a descriptive review of the scientific literature examining use rates of modalities and procedures used by CAM clinicians to manage chronic LBP and other conditions

**Data Sources:**

A literature of PubMed and MANTIS was performed using the key terms *Chiropractic; Low Back Pain; Utilization Rate; Use Rate; Complementary and Alternative Medicine*; and *Health Services *in various combinations.

**Data Selection:**

A total of 137 papers were selected, based upon including information about chiropractic utilization, CAM utilization and low back pain and other conditions.

**Data Synthesis:**

Information was extracted from each paper addressing use of chiropractic and CAM, and is summarized in tabular form.

**Results:**

Thematic analysis of the paper topics indicated that there were 5 functional areas covered by the literature: back pain papers, general chiropractic papers, insurance-related papers, general CAM-related papers; and worker's compensation papers.

**Conclusion:**

Studies looking at chiropractic utilization demonstrate that the rates vary, but generally fall into a range from around 6% to 12% of the population, most of whom seek chiropractic care for low back pain and not for organic disease or visceral dysfunction. CAM is itself used by people suffering from a variety of conditions, though it is often used not as a primary intervention, but rather as an additional form of care. CAM and chiropractic often offer lower costs for comparable results compared to conventional medicine.

## Background

Low back pain (LBP), especially in its chronic form, is a significant and continually growing health care problem for which the public seeks a great deal of expensive and potentially risky care. Recent research on the popularity and perceived effectiveness of complementary and alternative medicine (CAM) and integrative medicine for the treatment of both neck and LBP indicates that the public uses these forms of care in larger proportions than they do conventional medical care [1]. The effective and appropriate integration of CAM approaches with conventional medical interventions may be significantly enhanced and best accomplished with clear and concise evidence-based recommendations for the use of various CAM procedures and approaches to low back pain management. Ten years ago, the 1994 Agency for Health Care Policy and Research guidelines on back pain recommended the use of spinal manipulation as one important treatment option [2]. Since that time a great deal of new research information has been added to the scientific literature and this suggests to us that it is time to revisit those recommendations and to revise, update and reconsider them. Furthermore, other CAM modalities must, by necessity, also be considered, as should exercise therapy.

This paper is part of the second phase of a project addressing the long-term goal. The first phase was the development of a best practice document in concert with the Council on Chiropractic Guidelines and Practice Parameter; that effort is currently under review by the chiropractic profession. The long-term goal of this study is to review the existing literature, update and develop new and newly revised evidence-based best practice recommendations for the treatment and management of chronic and episodic LBP by chiropractors and other CAM practitioners. One of our specific aims was to compile a narrative review of the scientific literature to determine use rates of modalities and procedures used by CAM clinicians to manage chronic LBP and other conditions.

## Methods

A literature search was run using key terms *Chiropractic; Utilization Rate; Low Back Pain; Use Rate; Complementary and Alternative Medicine*; and *Health Services *in various combinations; this search covered the time frame from 1966–2005. Databases involved included Index Medicus, MANTIS (Manual, Alternative and Natural Therapy Index System), the Index to Chiropractic Literature, and CINAHL (Cumulative Index to Nursing and Allied Health Literature). Our goal was to cast a wide net and capture those papers that addressed CAM utilization in an attempt to provide an overview of the existing literature on this topic.

This yielded a total of 137 papers, which broke down thematically into 5 groups: back pain papers, general chiropractic papers, insurance-related papers, general CAM-related papers; and worker's compensation papers (of which there was only a single paper). The following sections of the report will summarize what the authors felt were the important findings from the papers we reviewed. While the papers yield a mix of research and other forms of information, no attempt to rate these papers in systematic manner was undertaken. Rather, the information here can be used to address the question of what the literature tells us about the use of chiropractors in delivering health care, what percentage of the population seeks chiropractic and/or CAM, what utilization rates are for LBP and other conditions, and what kinds of other conditions are seen by CAM practitioners. The scope of this descriptive literature review is broad and varied.

## Results and discussion

### Back Pain

Our primary intent in examining this literature was to investigate the modalities and procedures used by CAM clinicians to manage chronic LBP. Each of the papers discussed in this section provides one piece of a larger puzzle, demonstrating use rates in various settings and locations. Table 1 summarizes the results for the papers relating specifically to LBP, and also includes information on design and response rates.

**Table 1:** Table of results for back pain papers.

**Author**	**Reference**	**Design**	**N/Np**	**Main Results**
Hurwitz	5	Random sample of chiropractors from 6 sites	185/131 (70%)	68% of charts documented care for LBP; SMT was documented in 83% of charts. Chiropractic use rate has doubled in the past 15 years.
Shekelle	6	Analysis of insurance claims forms from 6 sites	5279	Visit rate for chiropractic was 41 per 100 person-years and rate of use of 7.5%
Cote	8	Mail survey	2184/1131 (55%)	People seeking care for back pain have worse health care status than those who do not.
Kelner	9	Interviews	300	87% of chiropractic patients sought care for LBP, with 77% believing their health care problem was serious in nature.
Walker	10	Mail survey	1768/1913 (69.1%)	55.5% of respondents with LBP in past 6 months did not seek care for it. Increased care seeking was associated with greater pain and disability, fear of pain impacting future activities, and female sex.
Sherman	11	Telephone interviews	249	Chiropractic was used the highest percentage of patients (54%); chiropractic patients had the highest rate of treatment-related discomfort of all groups.
Caswell	12	Self-reporting questionnaires	150	36% of the conventional therapy group had used at least 1 CAM therapy, while 62 of people in the CAM group had used conventional care. The higher the sociodemographic group, the likelier you were to use CAM.
Sundararajan	13	Prospective cohort study	1580	Factors associated with seeing multiple providers included presence of sciatica, higher Roland-Morris score, days to functional recovery and duration of pain prior to first visit.
Scheumier	14	Retrospective/prospective observational study	194 retrospective; 344 prospective	There was a substantial shift of referrals to manipulation practitioners under the scheme. Prospective patients had fewer referrals to secondary care, less drug use, and fewer certififed sickness days. Chiropractors used more x-ray than other practitioners.
Jamison	15	Mail survey	820/230 (27%)	Referral for visceral conditions met with little support; referral for LBP with frequent support
Leboeuf-Yde	16	Patient interviews by chiropractors	96/66 (66%)	82% of patients sought care for LBP; few sought care for visceral problems; most patients had short-term problems.
Cherkin	17	Random sample survey	Acu: 217/133 (61%) Chiro: 205/130 (63%) MT: 226/126 (56%) Naturo:170/99 (58%)	For chiropractic: woman made up 60% of visits; children 4%; older folks 20%; African American and Hispanic <10%; 80% of visits were by self referral; DCs provided equal amounts of chronic and acute care; back symptoms most common reason for seeking care.
Feuerstein	19	Analysis of National Medical Expenditure Studies		Percentage of people receiving chiropractic care was lower in 1997 compared to 1987, while percent of those receiving physical therapy grew.
Mayer	22	Mail survey	450/158 (35%)	~75% of chiropractors use 6 or more exercises for treating patients with LBP
Whitman	23	Interviews	131	There was a significant interaction between time and specialty certification status, but this disappeared on regression analysis.
Smith	24	Claims data analysis	9314 care episodes	Total payments within and across episodes were much greater for medically initiated episodes compared to chiropractic ones.

Noting that over $2.4 billion was spent on chiropractic services in 1988 [3] and that 19% of respondents to a survey about the use of unconventional medicine in the US sought chiropractic care [4], Hurwitz et al [5] examined the demographic and clinical characteristics of chiropractic patients as well as looked at visit rates in 6 sites located in the US and Canada. They chose 5 sites in the United States (San Diego, Portland, Minneapolis-St. Paul, Miami and Vancouver, Washington state) and one in Canada (Toronto) as representing a range in options with regard to geography, chiropractor-to-populace ratio and scope of practice laws. Site-specific chiropractic visit rates were calculated by multiplying the average number of visits for each chiropractor sampled by the number of chiropractors at that site and then dividing by the total population.

Hurwitz et al found that 68% of all visits were for LBP, and of these visits, more than 25% were related to sprains and strains; interestingly, no specific anatomical diagnosis was recorded in nearly 20% of cases. Of these patients, 45.4% had pain that had been present for less than 3 weeks, while 21.2% had pain that had lasted more than 6 months. Two percent had previous surgery for LBP, and for all patients just under 33% had sought care for their problems. That care had originally been delivered by other chiropractors, as well as general practitioners, orthopedists and physical therapists, among others. In the United States, the chiropractic visit rate was 101.2 visits per 100 person-years. This rate is far higher than in previous studies, which had seen rates of 41 per 100 person years [6] and 62 per 100 person years [7].

In examining who seeks care and where they seek it, Cote et al [8] tried to differentiate between those who sought care for LBP and those who did not. In doing so, it allowed them to look at the utilization for both neck and back pain. Here, a survey was conducted in Saskatchewan, Canada, with the sample drawn from 2184 subjects who were randomly selected. Of these, 1311 (55%) ultimately responded. They investigated 10 explanatory variables: demographics, socioeconomic variables, health-related quality of life, co-morbidity, neck and LBP, depressive symptomatology, cigarette smoking, anthropomorphic variables, exercise and previous neck or back injury. The survey also asked whether or not the responder had seen a health care professional for back pain in the last 4 weeks, and if so, who did they see? In the study, 907 patients reported either neck pain, back pain or both, and 15% of the total had chronic LBP. Fourteen percent sought medical care, while just over 12% sought chiropractic care. For those with pain who sought care in the past 4 weeks before the survey, nearly 30% sought chiropractic care alone (just over 31% sought medical care, and nearly 8% sought care form both MD and DC).

Kellner and Wellman [9] also looked at Toronto, Canada to examine the users of 5 modes of therapy: chiropractors, acupuncturists, naturopaths, Reiki practitioners, and family physicians (who are used as a baseline group). The authors found that in certain ways the chiropractic group differed strongly from the other 4 groups: a more equal sex distribution, a higher level of education, a higher level of household income and a higher level involved in full-time employment. Eighty-seven percent of the chiropractic patients had sought care for a musculoskeletal health problem, with 77% regarding their health problems as serious in nature. Interestingly, 93% also consulted regularly with their family practitioner.

Walker and colleagues [10] examined Australian adults seeking care for low back pain, using a population-based mail survey. The questionnaire provided cross-sectional data that looked at past and current status of LBP, involving disability, prevalence, who respondents sought care from and demographic information. Respondents were stratified into 3 age groups: 18–19 years of age, 20–80 years of age, and older than 80 years of age. Other variables included socioeconomic status, smoking status, lifetime physical fitness, lifetime emotional distress and fear of LBP, among others. A total of 2768 respondents were eligible, and 1914 returned questionnaires (with one rejected) for a response rate of 69.1%. Among its findings were that nearly 65% of respondents had at least one episode of LBP in the last 6 months, with the largest percentage reporting grade I pain. Of those with low back pain, 44.5% sought care, representing 28% of the complete sample. The most frequent types of practitioners seen were general practitioners (22.4%) and chiropractors (19.1%); while 41% of those seeking care in that time period sought it from a single practitioner, 59% received it from more than a single type of practitioner. Of those seeking chiropractic care, most were married, had educational levels that allowed them to work in basic or skilled jobs, were employed full-time (though just marginally higher than 50%), and were based in a large city (with the rest equally distributed among small and medium cities and rural areas). Those with LBP or fearful that their LBP could affect their life were more likely to seek care; women were more likely than men to do so as well.

Shekelle and Brook [6] analyzed data from the RAND Health Insurance Experiment (HIE) to derive population-based information on the use of chiropractic services. Here, the authors examined insurance claim forms for all fee-for-services patients who saw a chiropractor for care. They examined services provided and patient-specified symptoms, and derived population-based use rates per site. Use rate and services were calculated for both first and repeat visits.

In this study, 5279 people were enrolled in the HIE, representing 19021 person-years of exposure; 395 different people sought chiropractic care (7.5%), accounting for 7873 total visits (41 visits per 100 person-years). Repeat visits accounted for 82% of all visits; less than 1% of visits arose from referral from another health care provider. The most frequent reason for care seeking was pain, swelling or injury to the back (42%); manipulation accounted for the majority of services provided (39% at first visit, 60% in repeat visits). Overall, the study demonstrated a visit rate for chiropractic of 41 per 100 person-years and rate of use of 7.5% in a 3–5 year period. This is lower than in other studies, though this is also older data than in other studies.

Sherman et al [11] investigated the kinds of treatments patients were willing to try. Though their interest was in understanding what kinds of therapies patients interested in entering clinical trials might be willing to consider, the results may potentially be generalizable to other populations. The study was based upon the results of 249 patients who were willing to participate in telephone survey over a period of 7 months in 2001. The patients were located in both Washington state and Boston, Massachusetts, members of a non-profit health management agency. All suffered from chronic LBP. Of the therapies that were studied, the patient knowledge of each was rather low except in the case of chiropractic. Further, chiropractic had been used by the greatest percentage of patients (54%), with only massage close in comparison (38%). Knowledge and use of acupuncture, T'ai Chi and meditation were lower. Those who used the services of chiropractors also noted treatment-related discomfort or pain more than other groups. And most surveyed indicated they would be willing to use any of these alternative therapies if they were included in their health care plan and did not require any additional out-of-pocket expense.

On the other hand, Caswell and West [12] focused on the reason why people use less complementary therapies in Great Britain. Surveying 150 subjects, they collected data on knowledge, health care beliefs and other potential influencing factors. The subjects came from three specific groups of 50, all suffering from chronic LBP: private and National Health Service funded out-patient conventional management methods; private complementary therapies (CT) methods; and healthy, non-user subjects. They found that 36% of the conventional therapy group had used at least 1 CT in managing their pain (and when subdivided, the greatest use was among the physiotherapy patients, at 60%); 62% of the CT patients had used at least 1 conventional therapy (and here, 75% of the chiropractic patients had done so). The higher the socioeconomic group the more likely respondents were to select a CT; more than twice the percentage of patients who used CT felt they knew a great deal about the CTs, when compared to those who sought conventional care (88% vs. 41%). However, chiropractic was viewed as less efficacious compared to conventional therapy or other the other named CTs; and, the primary reason people sought care from a CT practitioner was referral from a friend or dissatisfaction with conventional therapy. They offered as part of their conclusion that it appears that the most significant barriers to the use of CT were lack of knowledge and lower socioeconomic status.

Sundararajan et al [13] looked at multiple provider use in acute LBP. This was a prospective study that followed patients from initial episode of LBP to recovery or 6 months, whichever came first. The providers included in the study included primary care physician, chiropractor, orthopedic surgeon or HMO primary care physician.

Here, 79% of the patients saw only the initial provider of care for their LBP. Using logistic regression, certain factors were associated with the use of multiple provider types: presence of sciatica; higher Roland Morris score; days to functional recovery; duration of pain prior to first visit; and, satisfaction were among the factors. The adjusted rates for multiple provider use were 14% for the primary care provider, 30% for the orthopedic surgeon, 9% for the HMO primary care physician and 19% for the chiropractor; that is, if a person sought orthopedic surgical care, they were 30% more likely to see multiple providers. Costs in such situations were much higher than when patients stayed with a single provider type ($435 vs. $1121). The results suggest that systems that use gatekeepers (here represented by the HMO primary care physician) may limit access or use of other care.

Scheurmier and Breen [14] tested the purchasing arrangements for acute LBP that were recommended to the UK health ministers by their Clinical Standards Advisory Group (CSAG). The study tested the CSAG's recommendations in primary care, looking at cost implications and identifying relations between UK general practitioners and those who offer manipulation services (which are predominantly chiropractors). The CSAG recommendations were similar in nature to those offered by the Agency for Health Care Policy and Research (AHCPR) [2]. The main difference was that here there was a shift toward a primary-care, wherein GPs would manage those with acute pain, making referral to chiropractors or other practitioners where they felt it necessary. The main reason for referral was if chronicity threatened. The outcomes included wait time for first visit, sickness certification, number of consultations, drug use and costs, recovery time, X-ray use and cost of share. The study did demonstrate a significant shift of referrals to manipulation practitioners using the new scheme. New patients were referred far less than existing ones, and used less overall services. Use of the guidelines did seem to be associated with better outcomes.

Jamison [15] looked at the kinds of disorders for which medical professionals were willing to refer to chiropractors. In this study 820 physicians were surveyed by mail. She found that referral for visceral conditions had little support, not surprisingly; however, referral for musculoskeletal conditions was more frequent, with back pain the most common reason for referral among all groups tested. Headache also ranked highly, but consideration was given as to potential cause for the headache.

In Sweden, Lebouef-Yde et al [16] looked at patients and treatment characteristics. Here, 86 chiropractors each interviewed 10 consecutive patients; outcomes included age, sex, previous chiropractic treatment, duration of complaint, area and type of treatment and number of return visits. Again, most patients sought care for LBP (82%) as well as, interesting, for pain in the lower extremity (52%). Nearly all complaints were musculoskeletal in nature; almost none for visceral problems. A low number of treatments was the rule; the authors believe this may have been due to economic pressures. Most patients had problems of a short-term nature (generally present for less than one month). About 25% had a chronic problem. Chiropractic did not appear to be the first choice of treatment for many people, which might explain the level of chronicity that was seen.

In yet another study, Cherkin et al [17] attempted to describe the patients and problems seen by CAM practitioners. Using a random sample of the practitioner types for the study drawn from 4 geographically unrelated states, data was collected on 20 consecutive patient visits. Data collected included demographic information, smoking status, referral source, reason for visit, concurrent care, payment source and visit duration; comparative data was drawn from the National Ambulatory Medical Care Survey [18]. In the case of chiropractic, the data indicated the following: Children comprised less than 4% of visits, but woman made up more than 60% of visits; older folks made up 20% of visits; visits by African-American and Hispanic patients comprised less than 10% of visits; 15% did smoke; 80% of visits were a result of self-referral to the chiropractor; chiropractors provided equal amounts of chronic and acute care, and provided care not related to illness in 12% of visits (likely representing wellness care); The most common reason for seeking chiropractic care included back symptoms (44%), neck symptoms (22%), wellness (10%), headache (4.6%), and shoulder symptoms (3.4%). Of the 4 groups in the study, chiropractic visits took the shortest amount of time (about 15 minutes). With regard to insurance coverage, the chiropractic rate ranged from 50–68% depending on the state. The authors note that chiropractors see a more limited range of conditions compared to acupuncturists and naturopaths, generally related mainly to musculoskeletal conditions. Another paper from this group examined the characteristics of the provider: acupuncturists, chiropractors, massage therapists and naturopaths [17]. In this study, random samples of each provider type were interviewed. The study found that a high proportion of practitioners were white, and were more likely to practice solo; few practiced with medical physicians. Chiropractors saw about 3 patients per hour and about 100 patients per week on average.

The use of non-operative care for treating LBP is growing and changing. Feuerstein et al [19] examined national trends regarding this by looking at data drawn from the 1987 National Medical Expenditure Survey and the 1997 Medical Expenditure Panel Survey. They looked at changes in rates of health service for back pain and occurrence of provider-specific care and type of service provided. The notable finding with regard to chiropractic care was that the proportion of individuals receiving chiropractic care was lower in 1997 compared to 1987; however, the proportion of those receiving physical therapy grew, from 5% to 9% in that same period. This demonstrates one of the growing challenges facing the chiropractic profession today; that of inroads being made into musculoskeletal management by other health care professions.

Weiner and Ernst [20] reviewed CAM therapies for the treatment of musculoskeletal pain in older adults. They call this review a "critical review" though they do not provide any indication of how the obtained papers for the review, nor how they extracted data. And further, they self-reference their own work in making significant criticism of the chiropractic profession and its treatment strategies for LBP, in that they discuss side effects of manipulation and quote their own work to support their contention that the reported incidence of side effects is probably too low; yet, the work they cite is yet another review, which they themselves wrote [21]. The authors conclude, on the basis of this analysis, that the benefits of spinal manipulation have not been shown to outweigh the risks, yet this is something (risk) that they have not actually studied.

It is important to note that chiropractors use treatments other than spinal manipulation in managing LBP. Exercise is often an important part of therapy. Mayer et al [22] examined chiropractors' patterns of use and perceptions of educational quality regarding exercise for LBP. Here, a questionnaire was mailed to a random sample of 450 chiropractors. The survey asked chiropractors to indicate which exercises they regularly were using for treating LBP, as well as the quality of the education they had received to prepare them to use those exercises. About three-quarters of the chiropractors surveyed used 6 or more exercises, with stretching/flexibility and abdominal strengthening exercises used more frequently. The study indicated that the use of exercise correlated well with how well the individual doctor felt they had been educated regarding that exercise.

Does experience or specialty certification status affect LBP outcomes? This is the question that Whitman et al examined [23]. Though only 13 therapists participated, the results were rather surprising: a significant interaction between time and specialty certification status was found for the manipulation group, but on regression analysis while the intervention group contributed to explaining the outcome, the therapists characteristic did not. That is, increased experience and specialty status did not appear to result in an improvement in the outcomes studied here. Some of the explanations offered for these results include the fact that clinical decision making was removed from the treatment of the patients in this study. Therapists could not choose the intervention that they were to apply, so another interpretation of the results is that the less experienced therapists were simply as able as the experienced ones to follow the study treatment protocols and follow the instructions. Also, this was a secondary analysis of data, so no randomization of therapists was possible.

Smith and Stano [24] did a retrospective analysis of LBP episodes, examining claims data from beneficiaries in the private-fee-for-service sector. Outcomes here included total insurance payments, total outpatient payments, length of initial and recurrent episodes, and time lapsed between episodes. There were 7077 patients represented, within 9314 episodes of care. From this, they found that total insurance payments within and across episodes were much greater for medically initiated episodes, that chiropractic providers retain more patients for subsequent episodes but that there is no difference in lapse time between episodes for chiropractic vs. medical providers. Chiropractic patients had a higher level of chronic cases in its mix.

Another study [25] used the National Ambulatory Medical Care Survey [18], in this case one looking at the osteopathic profession and asking whether or not it demonstrates its unique characteristics. The authors compared the practice patterns of osteopathic and allopathic physicians in the management of MS conditions in the family practice setting. The general results indicated that the osteopaths spend more time with their patients, had more injury-related visits that were self paid, provided more manual care and CAM therapy, and had fewer minority patients than medical practitioners did. Medical physicians ordered a greater number of diagnostic tests than did the osteopaths.

### Utilization

Many questions have been examined with regard to chiropractic and CAM use by the public. Table 2 summarizes the results of the papers specifically relating to utilization, and also includes information on design and response rates.

**Table 2:** Table of results for utilization papers.

**Author**	**Ref**	**Design**	**N/Np**	**Main Results**
Simpson	26	Mail survey	1509/784 (52%)	52% response rate. Referral rates varied depending on type of practitioner being referred to: 95% of respondents would refer to a PT, but only 14% would refer to a chiropractor. Respondents did not feel chiropractors should have primary contact status.
McCann	28	Analysis of Medical Expenditure Survey Data	25096	Chiropractic comprised 6% of all office-based health care visits; chiropractors accounted for 14% of all non-physician office-based expenditures.
Sharma	29	Prospective longitudinal non-randomized practice-based observational study	1414	Patients who self referred to DCs were likely to be older and have higher incomes than those who self referred to MDs; those who expected to self-pay more were more likely to self refer to the DC.
Jain	30	Mail survey	1680/601 (36%)	Response rate 35.8%; Variables that predicted disuse of CAM included male sex, good health, lack of physician support for CAM, and belief that CAM is not effective. For chiropractic, presence of perceived side effects was a major reason for disuse.
Pirotta	31	Mail survey	800/488 (64%)	Less acceptance for chiropractic compared to acupuncture, hypnosis and meditation. 75% felt chiropractic was sometimes harmful, but 29% would refer to a DC.
Astin	32	Review	25 surveys	Chiropractic had second highest rate of physician referral (40% behind massage (43%). 53% believed in the efficacy of chiropractic.
Goldszmidt	34			58% MD referral rate to chiropractors
Verhoef	35			83% MD referral rate to chiropractors
Perkin	36			34% MD referral rate to chiropractors
Andersson	37			50% MD referral rate to chiropractors
Wharton	38			51% MD referral rate to chiropractors
Marshall	39			2% MD referral rate to chiropractors
Hadley	40			27% MD referral rate to chiropractors
Reilly	41			20% MD referral rate to chiropractors
Berman	42			56% MD referral rate to chiropractors
Borkan	43			15% MD referral rate to chiropractors
Cherkin	44			57% MD referral rate to chiropractors
Goldstein	45			51% MD referral rate to chiropractors
Hawk	46	Mail survey	1896/563 (30%)	68% believe chiropractic is a therapeutic modality; 82% believe it is complete system.
Berman	47	Conference survey	180/295 (61%)	70–90% consider complementary medical procedures as legitimate.
Smith	48	Mail survey	1877/815 (43%)	Chiropractors offer a substantial amount of care to those in underserved populations.
Eisenberg	49	Telephone survey	1991: 1539 1997: 2055	CAM use grew from 33.8% to 42.1%, but chiropractic grew only from 10.1% to 11%. Mean number of visits fell from 12.6 to 8.9.
Eisenberg	50	Telephone survey	2055/831 (40%)	70% of patients saw a medical doctor before seeing a CAM provider; 15% saw a CAM provider before seeing an MD.60% did not disclose their CAM use to their MD.
Kessler	51	Telephone survey	2055/831 (40%)	30% of pre-baby boom group had used CAM; 50% of the boom group had used CAM; 70% of the post-baby boom group had used CAM.

Simpson [26] looked at referral patterns among Queensland medical practitioners to chiropractors, osteopaths, PTs and naturopaths. He received a 52% response rate out of 1509 mailed questionnaires, with the notable finding that referral rates varied depending on the type of practitioner being referred to. Physiotherapists received by far the best reception, with 95% of respondents endorsing physiotherapy and an equal percentage feeling that they could do so in a hospital setting. Only 14% endorsed chiropractors, and only 23% endorsed referral to members of the profession. This was, however, a higher percentage than osteopathy (10%) or other (8%). By and large, respondents felt that PTs were legitimate health care providers within the health delivery system, but few felt that chiropractors were. Further, they did not feel that chiropractors should have primary contact status, nor should receive referral under the Worker's Compensation program in Australia. This survey illuminated a lack of support for a recommendation made some years earlier by the Australian Medicare Benefits Review Committee [27], which had suggested "that upon application, the Commonwealth fund on a salaried or sessional basis, a limited number of appointments of chiropractors in public hospitals and/or community health centres or clinics." This paper suggests that work must be done to aid other professions in understanding what chiropractic is about and how one would determine what a competent practitioner looks like.

A paper by McCann et al [28] reported that chiropractic visits comprised about 6% of all office-based health care visits, and about 25% of visits to the offices of health professionals that were not MDs (in fact, their wording was "...to the offices of health professionals other than physicians," a point which should not pass without comment, since chiropractors are indeed physicians, just not allopathic ones. This suggests that chiropractors are not seen as "physicians."). Chiropractors accounted for 14% of "non-physician" office-based expenditures, and only 9% of visits were the result of a referral from a medical physician. By far, the greatest percentage of chiropractic cases involved back problems, sprains and strains (52%).

Sharma, Haas and Stano [29] asked about patient attitudes and other determinants of self-referral to chiropractors and medical physicians. They noted differences between patients who self-referred to medical physicians as opposed to those who self-referred to chiropractic physicians. Those who self-referred to the chiropractors were likely to be older and to have higher incomes, compared to those who referred to MDs; those who expected to self-pay were more likely to refer to the chiropractor, while those who expected their care to be paid by a third party payor chose the medical physician. Some seemingly mundane findings were that those who chose chiropractors were more likely to be opposed to prescription drugs, more likely to have confidence in chiropractic care, and had less disability. This information can help us understand utilization factors.

Jain and Astin [30] looked at barriers to access. The study population here was 1680 Stanford University alumni; 601 responses were received (response rate of 35.8%). Certain variables predicted disuse of CAM: being male; being healthy; lack of physician support for CAM; belief that CAM is not effective. The belief that CAM had significant side effects was related to low use of chiropractic in specific, as was lack of knowledge of CAM, a positive health status, and a lack of good office locations. Please note the low response rate and a non-representative sample, inasmuch as Stanford alumni tend to be white, affluent and highly educated.

Pirotta et al [31] asked if complementary therapies are accepted in general practice. This surveyed 800 Australian (Victorian) GPs, with a response rate of 64%. The survey revealed that there was wide-spread acceptance of certain CAM therapies, such as acupuncture, hypnosis and meditation, but less so for chiropractic. It is interesting to note that 8% of respondents stated that they have trained themselves in chiropractic, and 25% were interested in receiving such training in the future. Nearly 75% felt that chiropractic was occasionally harmful, and many felt that the placebo effect played a role in patient response. Twenty-nine percent said they would be willing to refer to a chiropractor.

Following up on the ideas presented above, Astin et al [32] looked at the incorporation of CAM by mainstream physicians. They examined 25 surveys which had been conducted between 1982 and 1995 studying the practices and beliefs of conventional physicians toward the 5 most prominent CAM therapies: acupuncture, chiropractic, homeopathy, herbal medicine and massage. Massage had the highest rate of physician referral, at 43%, while chiropractic was second at 40%. Rates of CAM practice by conventional physicians ranged from 19% for chiropractic (highest) to 9% for homeopathy (lowest). Fifty-three percent of the physicians believed in the efficacy of chiropractic. Physicians perceived chiropractic to be more useful and effective than acupuncture, which was itself seen as more effective that homeopathy. As they noted, this jibed well with the meta-analysis by Ernst [33].

Other studies [34-45] have shown referral rates by medical doctors to chiropractors that range from a low of 2% to a high of 83%.

CAM practitioners use other forms of CAM as well. Hawk et al [46] examined the use of CAM practice among a population of chiropractors. This random sample of 563 chiropractors scattered across the US showed, first, a schism in thinking in how to view chiropractic, with 68% of respondents believing that chiropractic should be viewed as a therapeutic modality, while 82% felt it should be seen as a complete system. More to the point, 72% used acupuncture, 72% massage, 63% mineral supplements and 56% used herbs for therapy. In comparing the results to those obtained to a set of medical doctors in the Chesapeake Bay area [47], some commonality was seen, as well as some disparity. For example, 14% MD vs. 17% DC used acupuncture, but 7% MD vs. 56% DC used herbs, and 31% MD vs. 8% DC used hypnotherapy. The largest disparity was with acupressure (13% MD vs. 72% DC) and biofeedback (54% MD vs. 14% DC).

One can also ask about the roles chiropractors play in the greater health delivery system. Smith and Carber [48] examined the use of chiropractic health care in health professional shortage areas. Their essential finding was that chiropractic providers offer a substantial amount of care to underserved populations, especially in rural areas. Practice volumes tended to be higher in these regions, and these practices may evolve as a result of patient needs in those areas. Certainly, it is likely that people in such areas might seek chiropractic care as their first point of access to the health care system, especially if the chiropractor is the sole provider for many miles.

Eisenberg [4] has added to his original report on the use of what he then called "unconventional medicine." His update in 1998 [49] showed that the use of alternative therapies continued to rise over the previous study, from 33.8% in 1990 to 42.1% in 1997. Chiropractic rates had only a modest rise over that time, from 10.1% to 11.0%, but the percent that saw a patient in the last 12 months rose from 71% in 1990 to nearly 90% in 1997. However, the mean number of visits fell from 12.6 to 8.9 over that same period. Overall, however, alternative medicine use and expenditures increased over the time from the last study to this one. Eisenberg followed this study up with one that looked at perceptions about CAM relative to conventional therapies [50]. This survey included 831 adults who had sought both medical care and used a CAM practitioner during 1997. The results demonstrated that 70% saw a medical doctor before seeing the CAM provider, and 15% saw the CAM provider before the medical provider; however, confidence in either type of provider was similar. Over 60% of those in the survey did not disclose their use of CAM therapy to their medical doctor, for a variety of reasons. In looking at case management, respondents felt that CAM was more effective for back pain, neck pain and headache than medical care, but that medical care was more effective than CAM for hypertension. What is of note here is that the results suggest that dissatisfaction is not a primary reason seek CAM. CAM has been growing in popularity, and the reasons may have more to do with belief that the interventions offer benefit in its own right. And further investigation of this work led Kessler et al [51] to find that the trend for the use of CAM continues, and will affect the delivery of health care into the future. In this survey, which in this case involved 2055 respondents, the use of CAM increased depending on the respondent placement relative to the so-called "baby boom." About 30% from the pre-baby boom cohort used some form of CAM, about 50% of those in the cohort itself used at least one form of CAM, and almost 70% of the post-boom cohort had used CAM. No one population sector showed a predominance of CAM use. The obvious point is that the use and acceptance of CAM has been growing over a period of many years and will likely continue to grow into the future.

### What Populations Have Been Studied?

Complementary medicine use has been examined in a variety of populations. Ernst and White [52] surveyed the use of CAM in the UK, finding that 20% of the population surveyed had used CAM in the previous year. Chiropractic amounted to less than only 3% of use. This level was so low that the authors speculated it might have been underestimated in the sample. The primary reason for the use of CAM was the belief that it is more effective and that people have a liking for it. A second study, by von Gruenigen et al [53] found that 36% of Amish women seeking obstetric care had used at least one form of CAM. Sixteen percent had used chiropractic care, which is a higher rate than reported in the survey by Eisenberg et al [4] looking at the general population, which found that the national rate was just under 12%. Yamashita et al [54] examined the issue in Japan. While nutrition rated very high (43.1% for both nutritional and tonic drinks and dietary supplements), it is interesting to note that the chiropractic rate was 7.1% in a country where the profession remains largely unregulated. However, 60% of those surveyed noted that the primary reason for the use of CAM was that their condition was not serious enough to warrant Western medical intervention. Eighty percent of those who sought chiropractic care did so for musculoskeletal problems, with just over 12% doing so for an undefined "other." Barnes et al [55] looked at use rates among US adults. Over 31,000 people were interviewed, and over 62% of those interviewed reported using at least one CAM therapy in 2002, the year of the study. In this study, the most common reason for the use of CAM was for back and neck problems, as well as joint pain and the common cold. This study found a use rate for chiropractic of 7.5%, with the highest modality being, interestingly, prayer for one's own health. Younger and older groups used CAM the least, while in terms of gender, women sought CAM care more frequently than men. Factor-Litvak [56] examined the use of CAM in women in New York City. The pilot study aimed to also look at racial and ethnic differences and for that reason included white, Hispanic/Latina and African American women. Three areas of CAM were studied: medicinal teas, homeopathic remedies, herbs and vitamins; yoga, meditation and other spiritual practices; and manual therapies. Chiropractors were the most frequently visited CAM practitioner (17%), while medicinals were the most frequently used category of CAM. There were little differences between the racial and ethic groups.

Smith and Carber [57] discuss the use of public-use survey databases that contain CAM information. This project helped to identify readily available public-use databases and datasets that can be tapped by health services researchers or others seeking utilization data. In doing so, the project developed a report that lists the surveys, and provides information on the sponsoring agency, survey objectives, sampling frames, methodologies used, time frame for data collection, chiropractic/CAM variables, information on how to locate and access the information and summary descriptive statistics. This report makes it possible for interested researchers to access useful data pertaining to CAM already in the public domain.

In examining the use of chiropractic in the rural health setting, Hawk and Long [58] analyzed the results from a set of 1511 survey respondents who were asked about their use of chiropractic services. The study found that just over 15% of respondents had used chiropractic services in the past year, with more than half doing so for the treatment of LBP (57%). Chiropractic use was more likely in the rural setting compared to non-rural settings; this makes common sense because in many rural areas chiropractic care may be the only care offered within the community. Chiropractic care was less likely in a variety of non-white populations: African American, Hispanic and Asian populations. Interestingly, chiropractic care was more common in Protestants compared to Catholics (outside of the state of Iowa). Over 42% of people with LBP used chiropractic care. Thus, a number of factors seem to affect the use of chiropractic care in the states studied (Illinois, Iowa, Minnesota, Missouri, Nebraska, South Dakota and Wisconsin). Also of interest, the period prevalence of chiropractic ranged from a high of 24% (Iowa and South Dakota) to a low of about 13% (Missouri, Nebraska). This cannot solely be attributed to the presence of a chiropractic college (Palmer College of Chiropractic) in Iowa, since there is also one in Missouri (Logan College of Chiropractic).

We are beginning to see chiropractic move into new settings. Dishman and Katz [59] discuss how a chiropractic clinic was established within a geriatric inpatient rehabilitation hospital. There are few such models in the profession, yet it is undeniable that such models will become more common as time goes on and information on utilization such as is demonstrated in this report becomes better understood. Integration of chiropractic into medical settings may be seen as selling out by some within chiropractic, as a move toward more primary care by others, and as a natural evolution by yet others.

Nelson et al [60] offer a commentary that addresses challenges to integration. They offer a number of issues they feel must be addressed in order for chiropractic to fully participate in emerging health care models: manual therapy diversity; research methodology; treatment of systemic dysfunction; and professional relations. With regard to manual therapy diversity, the authors note that we have a growing body of technique systems and techniques, and to try and define chiropractic just in terms of high-velocity low-amplitude (HVLA) fails to capture the full gamut of what we do. They then note problems with regard to research, such as the lack of comparative control group for those who receive an HVLA manipulation, lack of a sham and other simulated treatments, etc. They question whether or not chiropractic has a significant role to play in the treatment of systemic dysfunction. The treatment of systemic dysfunction has been declining [61] and little research attention has been focused in this area. Nansel and Slezak [62] present a reasoned review arguing against a chiropractic effect for visceral disorders. Finally, the authors ask what role we wish to play: primary care, portal of entry, specialist, generalist, etc.? We have yet to address this question definitively at the professional level, though the recent spine care paper by Nelson et al [63] is one attempt to do so. However, until we agree on what we are, we will find an impediment to our full integration.

Another paper addressing inter-professional collaboration studies the job satisfaction of chiropractors [64]. The idea of this paper was to look at how their relationships with medical doctors affects chiropractic job and career perceptions. In total, 311 chiropractors were surveyed. Results indicated that career satisfaction was related to satisfaction with compensation, how the chiropractor relates to his or her patients, and having good relations with other DCs. As has been seen in other reports, DCs reported referring far more patients to medical physicians than were referred to them by the MDs. The level of satisfaction with MDs is directly related to the referral rate; but overall, DCs' overall satisfaction rates with their career relies very little on their relationships with MDs.

Pirotta et al [31] ask whether or not CAM therapies have been accepted in general practice. This surveys Victorian general practitioner attitudes toward CAM as well as their use of CAM interventions. The survey was through the post, and 488 of 800 individuals responded (64%). The outcomes here were different than in other studies; the investigators looked at GP knowledge of CAM; opinions about effectiveness and harmfulness; appropriateness for practice; perceived patient demand; need for undergraduate education; referral rates, and training/practice in each therapy. In this survey, the most accepted forms of CAM were acupuncture, hypnosis and meditation, with over 80% of GPs having referred patients to practitioners of those therapies. Only 8% of the general practitioners claimed to have any training in chiropractic, but 24% were interested in obtaining training in chiropractic and other therapies. Still, many therapists felt that the placebo effect could explain the positive results seen in research studies. About 75% felt that chiropractic was occasionally harmful; but, about 55% considered it appropriate for trained GPs to practice. The respondents noted that the demand for CAM was increasing.

Gensler [65] offers suggestions of the place of chiropractors in North Carolina. This early study noted at its outset that most investigations of health personnel focus upon those practicing biomedicine rather than alternative care modalities, which at the time fell under the classification of ethnomedicine. He looks at chiropractic from several perspectives from the social science literature: system status, cultural congruence, and utilization patterns. With regard to system status, Gensler discusses how chiropractic had been seen as a deviant profession, then later as a low caste form of health care. This again changes, as when Coulehan [66] notes that chiropractic is no longer seen as politically or socially deviant. Chiropractic now has a constituency all its own.

With regard to cultural congruence, Gensler [65] again shows an evolution in thinking toward chiropractic. From Anderson's comment that the status of spinal manipulative therapy was shaped more by culture than by medical science [67] to chiropractic being seen a "natural," there is a change in the manner in which the public perceives how chiropractic is a part of the culture. McCorkle [68] describes how rural Iowans, who were mainly farmers, enjoyed understanding how chiropractic worked when it was presented to them using mechanical analogies, while Cleary [69] looks at how religion played a role in how culture and practice aligned.

And Gensler [65] also looks at what information on utilization was present at the time. In North Carolina proper, DCs had the lowest representation in those places with the greatest population and a far higher presence in areas with few people. DC/population ratios were correlated with lower incomes, something that is not the case in most modern studies.

When both chiropractors and medical physicians share patients, how well does communication occur between them? This is the question asked by Mainous et al [70]. This cross-sectional study included 400 DCs and 400 MDs, with a total 360 completing the survey (49%, 227 DCs and 133 MDs). The main finding was that though there is a high degree of interaction between the two practitioner types, MDs received information from chiropractors in 26.5% of referred cases, while DCs received information in 25% of the reverse referrals. Both felt they did not get enough information, and while both were not comfortable with sharing care, MDs were more uncomfortable than DCs. This suggests that much more can be done to help coordinate efforts between the two professions as they work on shared patients. It is important that communication between both groups be robust; otherwise, the risk increases that information collected by one practitioner may be overlooked or not heard by another, and this increases risk to the patient.

Table 3 summarizes the results from papers related to various populations, and also includes information on design and response rates.

**Table 3:** Summary table for population papers.

**Author**	**Ref**	**Design**	**N/Np And Population**	**Main results**
Ernst	52	Telephone survey	1204;British adults	20% had used CAM in the past year, with herbalism, aromatherapy and homeopathy ranking highest. Main reasons for use were perceived effectiveness and positive inclination toward it.
Von Greunigen	53	Survey	66; Amish women	36% had used CAM; 16% had seen a chiropractor in the past 12 months.
Yamashita	54	Telephone survey	1000; Japanese adults	Nutrition rated highest, at 43.1%; 7% of the population sought chiropractic care, in a country where the profession was unregulated. 80% of those seeking chiropractic care did so for musculoskeletal problems.
Barnes	55	Computer-assisted personal interviews	31044; American adults	62% used at least one form of CAM; 7% used a chiropractor, mostly for LBP.
Factor-Litvak	56	Computer-aided telephone interviews	300; women in New York City	Chiropractors were the most frequently visited CAM practitioners, at 17%.
Smith	57	Review of database source collections		Information presented here may allow researchers to access data on CAM in the public domain.
Hawk	58	Survey	1511	15% of respondents had used chiropractic in the last 12 months, with 57% doing so for LBP. Chiropractic use was higher in rural settings.
Konrad	64	Cross-sectional survey	467/311 (67%)	Career satisfaction of DCs was related to satisfaction with compensation, relations with patients, and good relations with other DCs.
Pirotta	31	Mail survey	800/488 (61%); Victorian GPs	Only 8% claimed to have training in chiropractic, but 33% were interested in obtaining training.
Gensler	65	Population distribution analysis from public data		DCs were associated with white populations and higher incomes.
Mainous	70	Cross-sectional survey	736/360 (49%) (227 DC and 133 MD)	MDs received information in 26.5% of referred cases, while DCs received information in 25% of referred cases; however, MDs felt more uncomfortable with this.

### Access and Insurance

Let us now turn our attention to access and insurance. How well is CAM being covered by insurance? Cleary-Guida et al [71] answer that question for one region in the United States, looking at New York, New Jersey and Connecticut. What they found in their telephone survey is that most insurers did cover chiropractic; what is not clear is the level to which that support is provided. Just less than half of insurers covered acupuncture; massage therapy was barely covered at all, and then only in conjunction with either chiropractic or physical therapy.

Metz et al [72] asked if chiropractic care was a substitution care or an add-on care in medical plans. Their paper analyzed claims data from a 4-yr period. The goal here was not to compare the costs of chiropractic vs. medical care, but rather to analyze the effect of a chiropractic benefit on the rates of patient complaints for a variety of pain conditions (i.e., back pain, neck pain) and on the number of episodes of care created by chiropractic and medical providers. They compared the rates of patient complaints in employer groups that both had and did not have a chiropractic benefit. Four cohorts were examined: (1) patients in health plans that covered chiropractic and who received any treatment (DC or MD) for a NMS condition; (2) Patients in plans that did not cover chiropractic and who were treated for NMS conditions; (3) Patients in plans that covered chiropractic and who received a chiropractic treatment for an NMS condition; and (4) Patients in health plans that covered chiropractic but who received medical treatment for NMS conditions. There were 3,129,000 patients with DC coverage and 5,197,000 without. Of this, 1,394,070 unique patients had neuromusculoskeletal system NMS conditions over the study period, of which 174,209 were chiropractic patients, 332,548 were medical patients with chiropractic coverage and 887,313 were medical patients without chiropractic coverage. The raw counts were then converted to rates per 1000 member years to allow for direct comparison of utilization between cohorts. The cohorts with chiropractic coverage had a rate of 162.0 complaints per 1000 member years compared to 171.3 for the group without chiropractic coverage. The authors conclude from this that patients use chiropractic care as a direct substitute for medical care, and not as an add-on.

Cost sharing is examined by Shekelle et al [73]. Here, the authors looked at data from the RAND Health Insurance Experiment, which was a randomized trial of the effect of cost sharing on the use of health services [74]. They found that chiropractic care was sensitive to price, with levels of coinsurance of 25% or greater leading to decreases in chiropractic expenditures by nearly 50%. Where there was access to free chiropractic care among those enrolled in HMOs, there was an increase in chiropractic use of about 9-fold; access to free medical care decreased fee-for-service chiropractic care by 80%.

Gordon [75] looked at CAM use by adults in a Californian HMO. She gathered information on prevalence of CAM modalities, how that prevalence varied by age and gender, and which modalities were increasing in popularity. The most common CAM modality was "prayer/spiritual practice you use yourself," followed by herbal medicine, massage, and then chiropractic (9.8%); however, for those who had musculoskeletal problems the use doubled to nearly 21%. More adults under the age of 64 used chiropractic than did seniors, but there was no gender difference in the population studied. As might be expected, having a chiropractic benefit was a predictor of its use; however, this was true only for men, not women.

Stano's 1993 paper [76] compared the health care costs for those who received chiropractic care for common NMS problems to those who were treated by either an MD or DO. This looked at 2 years of claims data (N = 395,641), based on 493 NMS ICD-9 codes. From this group, nearly 25% were treated by chiropractors, and those that did experienced lower health care costs in the fee-for-service sector; this was due largely to lower inpatient utilization. Total cost differences were found to be approximately $1000 over a 2-year period.

Work by Stano and Smith [77] that continued the original Stano paper [76] compared health insurance payments and patient utilizations patterns for episodes of care for low back problems treated by chiropractors or medical doctors. This study used 2 years worth of insurance claims data involving over 6000 patients who saw either a DC or an MD as their first-contact provider. MEDSTAT data was examined here; this is derived from fee-for-service claims information from large companies that have self-insurance plans. The authors used 9 trigger ICD-9 codes to initially identify the patients for the study, and note that though this is effective, it may not capture everyone with LBP. From this database, Stano and Smith found that chiropractors were the first-contact provider for about one-quarter of all first episodes and 30% of all episodes. After using multiple regression analysis to control for various factors (including differences in patient, clinical and insurance characteristics), they found that total insurance payments were much greater for episodes with medical first-contact care. The costs differences arose mainly due to higher inpatient payments for such cases.

Lind et al [78] also looked at claims data, so as to evaluate the prevalence and cost of CAM provider use for back pain treatment. Here, they analyzed outpatient claims for treating back pain using ICD-9 codes and provider type. They calculated the number of visits and expenditures for various forms of treatment. Four insurance products were identified for the study: health maintenance organizations, preferred provider organizations, point of service, and traditional indemnity groups. Provider types were categorized as being CAM (i.e., chiropractors, massage therapists, naturopaths and acupuncturists), conventional (i.e., medical and osteopathic), or "other" (i.e., occupational therapists, psychologists). They found that 57% of the study population had at least one outpatient visit to a conventional provider, while 55% had at least one visit to a CAM provider. The CAM visits accounted for 65% of all back pain visits, and of the CAM visits, about 75% were to chiropractors (accounting for almost 50% of all back pain visits). Specifically with regard to chiropractic, men were more likely to use chiropractors than women (OR 1.11, 95% CI 1.08–1.14); chiropractic care was highest in the smaller counties. In general, those who used CAM had more average visits, but had total resource expenditures that were lower than those who sought conventional care (average cost per outpatient visit: $50 USD, SD $28 vs. $128 USD, SD $173). Total outpatient costs were highest for the group that combined conventional and CAM care, while it was lowest for the group who used only CAM care ($1079 USD, SD $1185 vs. $342 USD, SD $429).

Thomas et al [79] looked at this question in the United Kingdom. They sent a postal survey sent to 5010 adults in England which focused on practitioner contact as well as the purchase of over-the-counter remedies. The researchers also gathered data on sociodemography, perceived health and National Health Service resource use (encounter expenditure, insurance and location of visit). The outcomes included population estimates of lifetime and last 12-month use for a series of CAM interventions, including chiropractic, acupuncture, homeopathy, and osteopathy. The response rate was 60%. Approximately 10.6% of the adult population had sought care from one of the CAM specialties during the last 12 months. All types of use declined in the older age groups. Chiropractic use in the last 12 months was 3.6%, and for lifetime use was estimated at 10.3%. Estimated total number of visits per year to chiropractors was 7.48 million, with a mean cost per visit of 22.80 pounds Sterling. This accounted for just under 160 million pounds per year for the estimated annual out-of-pocket expenditure.

Questions such as this have been looked at in less extensive settings. Phelan et al [80] look at utilization and costs for treating injured workers in North Carolina. The study examined, in addition to utilization and treatment cost, lost work days and compensation paid to those with MS injuries who treated either by an MD or a DC. Just under 100,000 claims were reviewed, of which 43,650 met the inclusion criteria. Out of these claims, just over 85% were treated by MDs, less than 1% were treated solely by DCs and just under 5% were treated by both. The average treatment cost for medical care averaged $3519, while the average cost for chiropractic care averaged $663. For those who were treated by both, the average cost for medical care was $4425 and the chiropractic care was $748, making a total of $5173. Compensation paid was over $17000 for patients treated by MD's, $3318 for those treated by DCs, and $23016 for combined care. The patients treated by medical care had a much longer time to discharge compared to those treated by chiropractors (176 vs. 33 days- and 240 days for those treated by both). It is possible that the longer time to discharge is due to more severe conditions in the medical group, but the authors note that one limitation to their study was a lack of data on severity of injury and comorbidity; however, this possibility has to be considered. Average total cost for claims was managed by MDs was over $25000, while for chiropractic it was just over $4000, with a total of over $33000 for combined care. Summarizing, the results show that services by DCs had lower treatment costs, fewer lost work days, lower compensation payments and lower use of ancillary medical services, though the use rates remain rather low.

A second paper looked at CAM use among rural North Carolinians [81]. This cross-sectional study looks at data from 1059 adults residing in Appalachian North Carolina. This grew out of a project known as the Mountain Accessibility Project, which used a survey to assess health factors in 12 rural North Carolina counties. A stratified cluster sample of adults was used. The response rate was nearly 84% and interviews probed the responses. "Home remedies" was the most common response, at 45.7%, with "honey-lemon-vinegar-whiskey" as the most common home remedy (26%). Just under 9% of those responding used an alternative therapist, with chiropractor the most common, at 6.7%.

Gray et al [82] looked CAM use in health plan members in Minnesota. Here, a managed care organization was examined in a cross-sectional mail survey. Just over 5100 people were surveyed, and just over 4400 responded (86%). The survey looked at use of CAM, patient characteristics, health behaviors, and interactions with conventional health care. From this group, 42% reported using at least 1 CAM therapy. While the most common CAM used was relation techniques (18%) and massage (12%), chiropractic was used by 8% of those surveyed, with nearly 90% of those who used chiropractic reporting beneficial results.

There have been developments in the delivery of CAM. Sarnat and Winterstein [83] report on clinical and cost outcomes of an integrative medical independent provider association (IPA). In their study, incurred claims and randomized patient surveys were analyzed for patient outcomes, cost offsets and satisfaction, compared to normative values. The study range included all members enrolled within the IPA during a 4-year period from 1999–2002. The goal was to see whether or not primary care physicians specializing in a non-surgical, non-pharmaceutical approach, and who used CAM interventions integrated with medicine would achieve better clinical and cost outcomes compared to the physicians who used standard medical care alone. The results demonstrated that there was a 43% decrease in hospital admissions per 1000, 58.4% fewer hospital days, 43.2% fewer outpatient surgeries and 51.8% pharmaceutical cost reduction compared to the standard care. It should be born in mind that this was a nonrandomized longitudinal study, which could not obtain appropriate statistical probability analysis due to an inability to obtain industry actuarial data.

Hurwitz [5] extended his analysis out over a much longer period. He looked at the demographic and clinical characteristics of chiropractic patients and to examine use rates in 6 sites across the US and Canada. The sites studied were located in San Diego, California; Portland, Oregon; Vancouver, Washington; Minneapolis-St. Paul, Minnesota; and Toronto, Ontario in Canada. This allowed the sites to reflect different geographical regions containing varying ranges of chiropractor-to-population rations and scopes of practice. The study asked 181 chiropractors to participate; 131 agreed to do so (71%). The results showed that about 68% of cases were for LBP, with the remaining 32% spread over several different clinical problems, nearly all of which were musculoskeletal in nature. 83% of the patients received spinal manipulation. There were differences across geographic regions with regard to median number of visits for care of LBP, and the length of time of care for LBP averaged nearly nice as much as median length of care for the other conditions (29 days vs. 14 days). The overall chiropractic visit rate was 101.2 per 100 person-years in the US, and 140.9 per 100 person-years for Canada. The results demonstrated that the use rates, at least at these sties, for chiropractic care are higher than in past studies.

An important paper by Legorreta et al [84] examined the effect of systematic access to chiropractic care on the use of chiropractic resources in a managed care setting. This study looks at 4 years of claims data involving over 700,000 members who had an additional chiropractic benefit and over 1,000,000 who did not. The individuals with chiropractic coverage had lower annual costs compared to those without it ($1463 vs. $1671), and having the coverage led to a 1.6% decrease in total annual health costs, after controlling for the cost-saving effects associated with favorable demographic and medical risk factors. For those with back pain who also had chiropractic coverage, they achieved lower use of plain film radiographs, lower hospitalization and less use of MRI. They also had lower back pain-related costs ($289 vs. $399). It is important to note that the results here refer very specifically to members of a single plan, and it bears further work that this be broadened to other plans.

Thomas [85] examines the access to CAM in general practice in England. Specifically, she looks at 6 kinds of CAM: chiropractic, acupuncture, homeopathy, hypnotherapy, herbalism and osteopathy). One-thousand two-hundred and twenty-six general practitioners were surveyed by postal questionnaire; this represented 1 in every 8 general practice partnerships in England. The questionnaire assessed estimates of how many of the GPs offered access to CAM in-house or made referrals for NHS patients to CAM providers. Response rate for the survey was just over 78%. Of these, just under 60% provided access to CAM in one form or another. Twenty-one percent offered the CAM care by a member of their primary care team; 6% employed an independent CAM therapist; and 24% made referrals to CAM providers. Acupuncture and homeopathy were the most common providers (21.2% and 16.8% respectively); osteopathy and chiropractic combined accounted for 7.1%, most of which was via referral.

The smaller setting is of interest to Hansen and Futch [86]. Their study looks at the use of chiropractic services in the non-Medicaid membership of the Group Health Cooperative of Southern Central Wisconsin for the years 1993–94. Medicaid is a federal health care plan designed to provide health care to low-income individuals. They also randomly sampled 500 members about their satisfaction with chiropractic care. Just over 5% of members used the services each year of the study, with the greatest use rates among women aged 35–49. Over 95% of those responding indicated satisfaction with chiropractic care.

Stewart [87] examined use and satisfaction in the delivery of CAM, though not including chiropractic. The goal here was to determine health rates and costs associated with providing CAM services in 2 benefit plans, and to see the level of patient satisfaction for those 2 plans. This involved 1091 patients in both plans who used CAM services during a single month of 1997 in the state of Washington. In this study, only 1% of the covered individuals from the 2 plans used CAM services during this period, though the percentage was higher in the PPO plan (1.2%) compared to HMO plan (0.6%).

As noted above, CAM use has been studied in the Amish [53]. Sixty-six percent of the women had used at least one form of CAM, with diet/nutrition (N = 7), herbal medicine (N = 14) and chiropractic (N = 16) ranking highest.

There have been studies looking at CAM use among children and adolescents. Sawni-Sakand [88] looked at CAM use among children seen in a primary care clinic in suburban Detroit. In this report, the most common forms of CAM used were herbs (41%), prayer (37%), megavitamin therapy or nutritional supplementation (34.5%), folk remedies (28%), massage therapy (19%) and chiropractic (18%). Factors in families that used CAM included mother's age 31 or older, religious affiliation, parent born outside the US and parent use of CAM. For the child, factors included age greater than 5, pediatric visit for illness, regular medication use and the presence of a chronic problem. Wilson and Klein [89] studied the issue in adolescents. In this study, 54% of the adolescents had used at least one form of CAM, with the most common being massage (13.2%), prayer (13.1%), herbs (11%), vitamins (10.6%) and special exercises (10.1%). Chiropractic was used by 6.7%. Nearly 15% of the boys used some sort of performance enhancing product, though less than 1% of girls did. Factors associated with CAM use included time spent in school clubs, use of health care without parental knowledge and parental and friend use of CAM.

Table 4 summarizes the results from papers related to access and insurance, and also includes information on design and response rates.

**Table 4:** Summary table for access and insurance papers.

**Name**	**Ref**	**Design**	**N/Np**	**Main results**
Cleary-Guida	71	Phone survey	70/43 (61%); NY, NJ, CT	Most insurers cover chiropractic, but to what level is not clear.
Metz	72	Analysis of claims data	3,129,000 with DC coverage; 5,197,000 without	Cohorts with chiropractic coverage had a rate of 162 complaints per 1000 member years, compared to 171.3 per 1000 for the group without coverage; patients use chiropractic are as a direct substitute for medical care, and not an add-on.
Shekelle	73	Analysis of data from the RAND Health Insurance Experiment		Chiropractic care was sensitive to price; levels of coinsurance of 25% or more led decreases in chiropractic expenditures by 50% or more; free access to care increased chiropractic use.
Gordon	74	Mail survey	1996–15,777; 1999–15,985; CA	Chiropractic was the third most CAM used, at 9.8%, but this more than doubled when looking at CAM use for musculoskeletal problems, to 21%.
Stano	75	Analysis of claims data	395,461 patients with appropriate ICD-9 codes	About 25% of patients were treated by chiropractors; those that did experienced lower health costs in the fee-for-serve sector, due to lower in-patient utilization.
Stano	76	Analysis of claims data	434,763	DCs were first contact providers for about 25% of all first episodes and 30% of all episodes. Costs for episodes with first medical contact were higher.
Lind	78	Analysis of claims data	601,044/104,358 (17%)	55% had at least one visit to a CAM provider; 65% of CAM visits were for LBP; 75% of visits for LBP were to chiropractors.
Thomas	79	Mail survey	5010/2893 (58%)	10.6% had sought care form at least 1 CAM provider; use declined in older age groups; chiropractic use in the last 12 months was 3.6%, but lifetime was 10.3%. Estimated total number of visits to chiropractors in the last year 7.48 million.
Phelan	80	Retrospective claims review	43,650	85% of claims were treated solely by MDs, 1% by DCs and 5% by both. Average treatment cost for medical care was $3519, and $663 for DC care alone; the combined group amounted to $4425 for the MD and $748 for the DC. Time to discharge for those receiving medical care was substantially longer than for the chiropractic care. Average total costs for claims was far lower in the DC group compared to the MD group or the combined DC-MD group.
Arcury	81	Mail survey	1059	Herbs, teas and other edible/drinkable remedy rated highest; chiropractic was used by 6.7% of the population surveyed.
Gray	82	Mail survey	5107/4404 (86%)	42% used at least 1 CAM therapy; chiropractic was used by 8%, and of those, 90% reported positive results.
Sarnat	83	Analysis of claims data	21,743	When CAM is integrated with conventional medicine, there is a 43% decrease in hospital admissions, fewer outpatient surgeries and reduced drug costs.
Legoretta	84	Analysis of claims data	700,000 with chiropractic benefits; 1,000,000 without	Those with chiropractic coverage had reduced annual costs compared to those without ($1463 vs. $1671); coverage led to a 1.6% decrease in total annual health costs.
Thomas	85	Mail survey	1226/964 (79%)	60% of those surveyed provided access to CAM; 21% offered CAM from another member of their team; 24% made referral to CAM practitioners (of which, 7.1% were referred to either a DC or a DO).
Hansen	86	Mail survey	500/191 (38%)	95% of those responding indicated satisfaction with chiropractic care.
Stewart	87	Comparison of benefit plans	1091	Only 1% of members used CAM during the study period, though the rate was higher in the PPO (1.2% compared to the HMO (0.6%).
Sawni-Sakand	88	Mail survey	1013; pediatric	Herbs and prayer used most, but chiropractic used by 18% of the study population.
Wilson	89	Telephone survey	1000/361 (36%); adolescents	54% used at least 1 form of CAM; massage most common (13.7%), and chiropractic at 6.7%.

### Specific Conditions

This section of the report addresses the use of CAM/Chiropractic in the management of specific conditions.

#### Older adults with Osteoarthritis

Ramsey et al [90] looked at the rates of use of expenditures for CAM therapies for adults suffering from osteoarthritis. The participants for this study were drawn from a group who were involved in a clinical trial of warm water exercise for osteoarthritis. They ranged in age from 55–75, and were located in the state of Washington. The participants recorded their use of both traditional and CAM services in a weekly postcard diary as well as in 2 far more detailed surveys administered at the beginning and end of the trial. Cost information was derived from a number of sources: Medicare reimbursement rates for CPT codes; averages for CAM care not covered by a CPT code were estimated by using average charges from a local survey of providers; medication costs were based on average wholesale prices; OTC medications were based on average charges from local pharmacies. The response was superb; 122 out of 124 people completed the study, 96% returned the weekly postcards and 99% completed the questionnaires. Fifty-eight individuals (47%) reported using at least 1 CAM therapy at the beginning of the observation period. Four percent used only CAM during the reporting period. The most common form of CAM was massage therapy, which was used by 57% of people, while chiropractic was second most common at 20.7%. The annualized expenditures for chiropractic (mean cost per user + SD) was $541.16 + $550.20, compared to $1422.65 + $1753.07 for massage therapy and $804.38 + $310.24 for acupuncture.

#### Breast cancer

VandeCreek [91] created a profile describing interest in and use of CAM available to breast cancer outpatients. They gathered data on the number of appointments for CAM therapies, costs, and reimbursement patterns; they then compared them to a published profile of the general public. The project used a survey to assess the patient's interest and use of CAM as well as to assess mental adjustment to the cancer experience and consequent personal growth from it. 112 patients participated. With regard to interest, the highest rates were seen for prayer (84.5%) and exercise (75.8%); after that, there was a drop to spiritual healing (48.3%), while chiropractic was at 13.8%. Approximately 2% of the breast cancer patients used chiropractors, which is perhaps not surprising given that this is on its face outside the normal scope of chiropractic conditions. It is also not surprising that prayer and spiritual healing would rate so highly in this class of patients.

Shen [92] specifically focused upon advanced-stage breast cancer. This study used face-to-face structured interviews of patients with advanced-stage breast cancer. One hundred fifteen patients were interviewed for the study, 84 of whom were users of CAM. When compared to non-users, these individuals were found to have higher education levels. Most used more than one form of CAM. The most common CAM product was herbal medicine, which was used by more than half of the CAM users, most of whom noted that this was not under the supervision of their primary health care provider. Many people used CAM in the belief that it helped to strengthen their immune system. Chiropractic was used by less than 10% of patients, and the reasons for that use were not explained.

#### Multiple Sclerosis

Looking strictly at naturopaths, Shinto et al [93] described the results of a survey about treatments and outcome measures used by NDs in managing patients with multiple sclerosis. The study found that 43% of those surveyed had treated at least 1 patient with MS; 68% communicated with the patient's medical doctor in rendering that care. The most common therapies recommended included diet (52%), essential fatty acid supplementation (44.6%), vitamin/mineral supplementation (33.7%) and homeopathy (30.7%). Early state patients perceived their treatment as being effective in 57% of cases, middle stage patients in 25.3% and late stage MS patients in 3.0%. Fifty-nine percent felt that the care helped improve their quality of life, while 48.2% felt that it helped decrease their relapse rates.

Work by Nayak [94] included chiropractic as part of the research. This was a postal survey of 11,600 individuals, of which 3140 returned the survey (response rate of 27.1%). More than half of those who did respond claimed to have used at least 1 CAM modality. The less satisfied with conventional care they were the more likely they were to use CAM. Ingested herbs (26.6%) and chiropractic (25.5%), along with massage therapy (23.3%) and acupuncture (19.9%) were most common in use. Women were 25% more likely than men to use CAM, and whites were 30% more likely to use CAM than non-whites.

There is one citation [95] to chiropractors treating MS in the literature indexed in PubMed but this is a single uncontrolled case report.

#### HIV

While no one recommends most forms of CAM as a stand-alone therapy, there are papers which examine the use of CAM by HIV-infected patients. Furler et al [96] looked at CAM use by HIV-infected patients attending an outpatient clinic in Ontario, Canada. This also allowed them to compare the users to the non-users. The study was a cross-sectional survey of a sample of HIV-infected patients. Among other inclusion criteria for the study was one that patients have a helper T-cell cluster of differential (CD4+) nadir of less than 500 cells per microliter. CAM use was assessed by patient report using a one-in-one, in-person, semi-structured interview, which was then coupled to a larger questionnaire. While the focus here was primarily on the use of vitamins, minerals and micronutrients, they did also gather information on specific forms of CAM. Overall, 77% of patients reported CAM use (nearly 90% if using a different definition of CAM). The most frequent reported categories of CAM included mind-body at 61.5% and use of vitamins at 57.7%. When combined and collapsed, nearly 89% of patients used some form of micronutrient. Just over 37% used a CAM provider, with massage coming in at 25% and chiropractic at 19.2%. The most common reason for using CAM was general well being, with relaxation, pain and stress also rating highly. Also, it should be noted that more than half of the people never reported the use of CAM to their primary medical physician.

In Bica's study [97], the location was Eastern Massachusetts and Rhode Island. This was also a cross-sectional analysis, using repeated measures from a cohort study, and using the study visit as the unit of analysis. There were 642 participants, who were surveyed for use of ingested and non-ingested CAM interventions. Nearly 60% of patients used some form of ingested CAM, while about 40% used a non-ingested form of CAM. Massage was most common (25%), while chiropractic was not included in the analysis.

Wiwanitkit [98] looks at CAM use of HIV-positive patients in Thailand. This was a survey of 160 HIV-positive patients, which found that 95% used some form of CAM, and 78% visited at least one CAM provider. Given the location, the most common form of CAM used was a visit for a ritual remedy from Buddhist temples. Again, chiropractic was not included among the therapies used.

#### Asthma

While there have been clinical trials of chiropractic for asthma [99,100], there is little information on use rates by asthmatics. Blanc's study [101] examined a random population telephone sample of 300 adults who self-reported a physician's diagnosis of asthma or rhinosinusitis without asthma. Results showed that 42% of participants used some form of CAM practice to help treat breathing or nasal symptoms at some point in the previous 12 months. Chiropractic was not specifically queried for in this study.

#### Cancer

Several papers have reported on the use of CAM by cancer patients. Ernst and Cassileth [102] offer a systematic review of CAM use for cancer. In their systematic review, they identified 26 publications, which demonstrated growth in this topic (1 paper from the 1970s, 9 from the 1980s and 16 in the 1990s to the date of publication). The review demonstrated that 50% of papers reported a use rate of up to 27%, with the rest showing that more than 25% of respondents used CAM. Percentages ranged from a low of 7% to a high of 64%, with an average of 31.4%. The most common CAM therapies used included mind-body approaches, reflexology, dietary approaches and food supplements.

Lewith [103] queried not just patients but staff as well. In this survey, 270 questionnaires were sent out and 162 responses were received. Here, 32% were receiving some form of CAM, with half of those receiving it being in hospice care. The most common forms of CAM included massage, nutrition, aromatherapy, relaxation and reflexology. The majority of those surveyed felt that CAM would offer palliative care, a few felt it could help cure their cancer. In surveying the staff, 486 questionnaires were sent out and 196 were received back. Twenty-one percent had some sort of CAM training, while nearly 66% would like to receive training. The interest levels were similar to those of the patients. In passing this report cites a short report by Rees et al which demonstrated that 6.4% of patients in the South Thames Region breast center had used chiropractic care [104], but this current paper did not assess chiropractic usage.

Similar work has been done for Turkish cancer patients [105]. In this study, 61% of patients used at least 1 form of CAM, with birthplace, educational status and family type significant factors for such behavior.

A study from Wales [106] looked at prevalence, cost and satisfaction with CAM. Here, 1697 patients were involved, with 1077 returning the survey (response rate of 64%). These participants had a cancer diagnosis of at least 3 months. Just about half of those surveyed used at least 1 type of CAM (49.6%) in the past year, 16.4% consulted at least 1 CAM practitioner and 15.4% used at least 1 form of CAM technique. The most common form of CAM was the use of over-the-counter diets, remedies or supplements (42.3%). CAM users in this study were more likely to be female, younger, better qualified and to have used CAM prior to their cancer diagnosis. CAM was used mainly for symptom relief and relaxation. The majority of patients were satisfied with the CAM they used. In this study, a total of 37 patients used chiropractic (3.4%).

#### Mental Disorders

Unutzer [107] performed a national survey to examine use of CAM by those with mental disorders. The survey itself was conducted in 1997–1998 and involved 9585 people. A set of screening interviews was used to establish diagnoses of probable mental disorder. In the sample, 16.5% reported use of CAM during the past 12 months, and 21.3% met the criteria for a diagnosis of mental disorder. Those with panic disorder and major depression were more likely to use CAM. However, chiropractic was not included in this analysis, because, in the words of the authors "this treatment is now covered by a large number of health insurers and most states have health insurance mandates to cover chiropractic."

The study by Kessler [108] looked specifically at anxiety and depression. This report was drawn from the same larger study that the paper by Unutzer [107] above did, but in this case the sample was 2055. Of these, 9.4% reported suffering from anxiety attacks, and 7.2% reported depression. Of these, 56.7% of those with anxiety attacks and 53.6% of those with depression used a CAM therapy in the last 12 months; however, only 20% of those with anxiety and 19.3% of those with depression visited a CAM therapist. In addition, nearly 66% of those with anxiety and 66.7% of those with depression sought care from a traditional provider. Chiropractic accounted for very small percentages, 0.5% for anxiety and 1.0% for depression.

How often do patients seek treatment for mental health problems by seeking CAM? Simon et al [109] address this question by sampling practitioners rather than patients. Disciplines included were acupuncture, chiropractic, massage therapy and naturopathy in 4 states. The proportion of visits for mental health problems ranged from 7–11% for acupuncture, massage and naturopathy to a low of less than 1% for chiropractors. The authors offer several explanations: that people with such problems are simply less likely to visit chiropractors; that those who do visit chiropractors may not mention a mental health problem over the course of their visit for another complaint; that the chiropractor is less likely to record mental health concern compared to the other practitioners. It is not possible to say.

Demling [110] surveyed psychiatric patients with regard to their use of non-medical alternative practitioners. There specific interest was in determining whether psychiatric patients consulted Heilpraktikers. Thus, 473 patients admitted to a university-based psychiatric hospital were surveyed; about one-third had consulted a Heilpraktiker. They had generally positive attitudes toward them, and felt substantial loyalty. For those with psychiatric complaints, about 11% used chiropractic methods, while about 17% used them for physical complaints. Homeopathy was, however, the kind of intervention used most frequently by the Heilpraktiker.

#### Special Needs Children

Children with special needs are defined as "those who have or are at risk for a chronic physical, developmental, behavioral, or emotional condition and who also require health and related services of a type or amount beyond that required by children generally" [111]. Sanders et al [112] found that 64% of the families in their survey reported using CAM for their child. The survey had a response rate of 82% (376/460). The most common form of CAM was spiritual healing/prayer/blessings. With regard to manipulation, children with cerebral palsy and spina bifida were more likely to receive this intervention; 4% visited a chiropractor in the last 6 months and 6% reported ever using chiropractic, while the rate for osteopathic manipulation was similar, at 3%/5%. Use of specific forms of CAM varied depending on what condition the child had.

#### Diabetes

A study that looked at gathering information from 4 separate high-risk groups for diabetes used the Summary of Diabetes Self-Case Activities Questionnaire to assess the frequency of CAM use and self-management activities [113]. The 4 groups included in the study were African Americans, Hispanics, Native Americans, rural whites. Twenty individuals from each group were interviewed and completed the above-named questionnaire. One in 4 of those sampled mentioned using some form of CAM for managing their diabetes, with the highest use among Hispanics (50%) and the lowest among rural whites (15%). Most CAM interventions were centered around holistic practices.

#### Emergency Department Visitors

Two papers describe the CAM use characteristics of patients who use CAM for reasons external to their need to seek emergency care [114,115]. Rolniak's paper [114] was a descriptive study of a convenience sample of 174 patients who came to the ER of a level 1 urban Catholic teaching hospital. In this work, CAM use was relatively high, with 47% of those who sought ER care using CAM, with prayer (28.2%), music therapy (10.9%) and meditation (10.3%) most common. Chiropractic was next, with a use rate of 5.7%. While it might be expected that visits to an ER should closely follow general demographics, this rate for chiropractic is slightly lower than the national average, which is just over 10% (48). Ba's study [115] was conducted in the emergency department of the University of California, San Francisco Medical Center. Here, the data indicated that, with regard to the presenting medical problem, the most common CAM used was herbs (7%) and spiritual healing (7%); chiropractic rated just 0.3%; however, when looking at who had used various forms of CAM in the past 12 months, 7% had seen a chiropractor, while herbs (24%) and massage therapy (17%) were most common.

#### Peripheral Neuropathy

A prospective questionnaire was used to survey 180 outpatients being treated for peripheral neuropathy [116]. Forty-three percent acknowledged use of CAM, with the most common forms being megavitamins (35%0, magnets (30%), acupuncture (30%), herbal remedies (27%) and chiropractic (21%). Just over one-quarter felt that the use of CAM helped improve their neurological symptoms, and the most common reason for using CAM was for pain control. About half of the patients who used CAM did so on their own, without consulting or notifying their medical physician, and most did not discuss their use of CAM with their primary physician.

#### Surgical Patients

A study by Wang [117] looked at differences in CAM use between in-patient and out-patient surgical patients. Their study indicated that more than half of patients (57.4%) used some form of CAM, with prayer (29%) and chiropractic (23%) most common. No real differences between the 2 groups were found, and both groups were largely unwilling to pay out of pocket for CAM, but were willing to accept it as part of their perioperative management. However, they were indeed willing to pay for chiropractic.

#### Primary Care Patients

This was surveyed in Israel and involved 480 patients from 2 primary care patients [118]. In this study, just over 18% of the respondents had used CAM, and most had used more than 1 form, with homeopathy by far the leading modality (34%). Reflexology was next at 18%; chiropractic was grouped in with "other" and received a rate of 16% en masse. However, the most common reason for seeking CAM was for musculoskeletal problems (22%). These results may represent accessibility for the particular region of Israel where the study was performed. It is also notable that there are few chiropractors in Israel.

Table 5 presents summary results for the papers discussing specific conditions, and also includes information on design and response rates.

**Table 5:** Summary table for papers discussing specific conditions.

**Name**	**Ref**	**Design**	**N/Np**	**Main Results**
Ramsey	90	Survey and diary	124/122 (98%); adults with osteoporosis	47% used at least 1 form of CAM; massage therapy used by 57%, and chiropractic used by 20.7%.
VandeCreek	91	Patient interviews	112; breast cancer patients	Highest rates for prayer (84.5%) and exercise (75.8%); chiropractic used by 13.8%.
Shen	92	Patient interviews	115; breast cancer patients	84 of 115 used at least 1 form of CAM, and those that did were found to be higher educated; many people felt it strengthened their immune system; chiropractic used by less than 10% of the population.
Shinto	93	Mail survey	927/385 (42%); MS was focus of study	43% of NDs had treated patients with MS; 63% communicated with the patient's MD; diet was considered important.
Nayak	94	Mail survey	11,600/3140 (27%); MS patients	More than half used at least 1 form of CAM; the more dissatisfied they were with conventional care, the more likely they were to use CAM. Chiropractic ranked high, with over 25% seeing a chiropractor.
Furler	96	Patient interviews	104; HIV patients	77% reported use of CAM, with mind-body ranked highest (61.5%) and chiropractic use rated at 19.2%.
Bica	97	Cross-sectional analysis using repeated measures from a cohort study	642; HIV patients	60% used some form of an ingested CAM, while 40% used a non-ingested form; chiropractic not included in the analysis.
Wiwanitkit	98	Interview survey	160; HIV patients	95% used some form of Cam, and 78% visited a CAM practitioner; chiropractic not included.
Blanc	101	Telephone survey	455/300 (66%); asthma patients	42% used some form of CAM within the past 12 months
Ernst	102	Literature searches	26 surveys; cancer	50% of papers showed a use rate of up to 27% (range: 7–64%; average of 37.4%).
Lewith	103	Patient questionnaire	270/162 (60%); cancer patients	32% received at least 1 form of CAM; this was felt to offer palliative care; massage was most common.
Ceylan	105	Patient questionnaire	326/305 (94%); cancer patients	61% used at least 1 form of CAM; significant factors included birthplace, educational status and family type.
Harris	106	Mail survey	1697/1077 (63%); cancer patients	Half of those surveyed had used at least 1 form of CAM; 16.4% consulted a CAM practitioner; and dietary interventions were most common. 3.4% had used chiropractic services.
Unutzer	107	Telephone survey	9585; mental disorders	16.5% had used CAM in the last year; those with panic disorder and depression were most likely to be users.
Kessler	108	Telephone survey	2055; anxiety and depression	56.7% of those with anxiety attacks and 53.6% of those with depression used CAM in the last 12 months. Chiropractic care accounted for less than 1% of CAM use in this survey.
Simon	109	Systematic multi-state study of CAM providers	Acupuncture: 2561; Chiropractic: 2550; Massage: 2005; Naturopathy: 1817; mental health	Proportion oif visits for mental health ranged from 7–11% for all but chiropractors, who ranked at less than 1% of visits for mental health reasons.
Demling	110	Patient questionnaire	512/473 (92%); psychiatric patients	About one-third had seen a Heilpraktiker; 11% used chiropractic methods.
Sanders	112	Parent survey of children undergoing treatment	460/376 (82%); special needs children	64% used CAM ; children with cerebral palsy or spina bifida were more likely to use manipulation; 4% had used a chiropractor in the last 6 months, while 6% reported using when at some time in their life.
Schoenberg	113	Patient interviews	80; diabetics	25% used at least 1 form of CAM, with Hispanics using it most frequently (50%).
Rolniak	114	Descriptive study	174; patients presenting to the ER	47% used at least 1 form of CAM; chiropractic was used by 5.7%.
Li	115	Patient questionnaire	356; patients presenting to the ER	At visit, only 0.3% had recently seen a chiropractor, but 7% had seen one in the past 12 months.
Brunelli	116	Patient questionnaire	180; patients with peripheral neuropathy	43% used at least 1 form of CAM; chiropractic was used by 21% of patients. Megavitamin was most common (35%).
Wang	117	Mail survey	Not reported; surgical patients	57.4% used at least 1 form of CAM, with 23% using chiropractic care.
Kitai	118	Patient questionnaire	480; Israeli primary care patients	18% used CAM, with most using more than 1 form; homeopathy was most common (34%0, with chiropractic lumped in with "other" at (18%).

### CAM in Specific Settings

This section of the paper will examine a number of papers studying CAM usage patterns in various settings and among various groups, not among those with specific conditions. A study by Cuellar et al [119] compared CAM use by African Americans and Caucasian Americans in rural settings. This involved a convenience sample of 183 elders from community service organizations in the state of Mississippi. Past work [120] has demonstrated that older folk do commonly use chiropractors as a significant part of using CAM. Here, the combined results from both groups showed that the most common CAM therapies were, in order, prayer, vitamins, exercise, meditation, herbs and chiropractic; however, what was notable was that there was a difference in chiropractic use between African Americans (7.5%) and Caucasian Americans (19%). African Americans do find certain barriers to seeking chiropractic care [121]; these included lack of knowledge of what the profession has to offer, limited awareness, and distrust of medical research due to past abuse such as Tuskegee (where black sharecroppers suffering from syphilis were kept in a trial without consent long after a cure had been found simply to study the long-term natural history of the disease). At present, we do not completely understand why this difference persists, though the scope of health care coverage offered to the middle class might include chiropractic, while those from a poorer economic status may need to use what health resources are available for them. It is also worth noting that the study took place in the so-called "Bible belt" of the United States, which may give reason as to why prayer ranked so high. Further, it is debatable whether exercise should be construed as CAM.

Lynn Keegan [122] examined the use of CAM among Mexican Americans near the Rio Grande Valley in Texas. A convenience sample of 213 Mexican American subjects was used. The most common forms of CAM used included herbal medicine (44%), prayer (29.5%), massage (28.3%), relaxation techniques (22.5%) and chiropractic (19%). Open-ended comments regarding chiropractic included "I go to chiropractors only for muscular pains," "seems to work temporarily," "very good, helps a lot" from men, and "The chiropractic therapy worked very well, I wish I could have continued going a little longer," and "Problems with back and neck pain, chiropractic does help."

Sirois [123] examined the treatment seeking patterns of CAM and conventional medicine users across different health problems, as well as the kinds of treatments used by those with varying levels of CAM experience. This project used a 3-group cross-sectional survey administered to 199 self-selected participants. There has been evidence found that suggests that CAM users have more major medical problems [124], report poorer overall health status compared to nonusers [125] and have more chronic problems. [126] Thus, it bears importance that some effort be made to better understand the differences in the characteristics of those who seek CAM compared to those using conventional medicine. In this study, 13 general physicians along with 4 CAM practitioners (including 1 chiropractor) were surveyed. Indeed, the health problems were highest for the established CAM (ECAM) clients, and lowest for the conventional medicine (CM) group. However, the ECAM group sought care for more non-life-threatening health issues than the CM group. More ECAM clients sought care for back problems than the CM group (61% vs. 19%). New users of CAM did not seek care for LBP in as high a number (53%), indicating that they might not have yet completely transitioned to full-time CAM use.

Given that so much information now exists, Harris and Rees [127] attempted to systematically review the data on prevalence of CAM use in the general population. The authors selected 2 databases for their study: Medline and the Centralized Information Service for Complementary Medicine (CISCOM) [128], and included papers is they used survey methods to estimate the extent of CAM in a target population, measured CAM use among the general population (as opposed to a clinical population) and estimated the prevalence of CAM as a percentage of the population. From this initial survey, 638 papers were uncovered (491 on Medline and 147 on CISCOM). Of these, only 12 met the study inclusion criteria. Most studies had at least one methodological flaw. The studies with the greatest rigor all demonstrated that the use of CAM in the USA is growing and is being used by a high proportion of the population.

What can be said about why women seek CAM practitioners? This is the question asked by Adams et al [129] in their survey of Australian women. This study involved nearly 42,000 people and was derived from the Australian Longitudinal Survey of Women's Health [130]. Participants completed an SF36, and were asked about the conditions they sought care for as well as the frequency of use of CAM over the past 12 months. In general CAM users had poorer health status than non-CAM users, and reported lower levels of physical functioning. CAM users also made more visits to general practitioners and to outpatient clinics.

Integration of CAM into conventional care is starting to take place. Giordano et al [131] offer thoughts as to how research may help foster integration of CAM into mainstream public health. Giordano notes a need for research to define mechanisms of CAM-based therapies (and indeed the National Institutes of Health has called for proposals specifically addressing this need) as an aid to integration. This can be addressed by training CAM clinicians to act as researchers, in order to develop pragmatic trials to provide credible evidence for the use of CAM in the public health setting. This can be done by developing programs in CAM educational and health care settings that provides such training; programs that should receive support from extramural agencies as well.

O'Brien [132] also notes the contributions that CAM may bring to the public health arena. While her focus is Australian and is with regard to optometry, she notes the growing focus on CAM from private health insurance companies, from governments and even from the World Health Organization. CAM is making inroads at every level, but has a number of emerging issues: safety of CAM practices; quality control, integration of CAM and standards of practice, its potential, and its evidence base.

Table 6 presents summary results for the papers discussing specific locations, and also includes information on design and response rates.

**Table 6:** Summary table for papers examining specific settings.

**Name**	**Ref**	**Design**	**N/Np; setting**	**Main Results**
Cuellar	119	Descriptive comparative study	183; African American and Caucasian elderly in rural settings.	Most common forms of CAM used were prayer, vitamins, exercise, meditation and chiropractic; there was difference in chiropractic use between African Americans (7.5%) and Caucasians (19%).
Keegan	122	Descriptive study	213; Mexican Americans in the TX Rio Grande Valley	Most common forms of CAM used included herbs (44%), prayer (29.5%), massage (28.3%), relaxation (22.5%) and chiropractic (19%).
Sirois	123	Self-selected patient questionnaire	199; CAM and Cm patients	CAM patients had the most health problems, but sought care for more non-life-threatening diseases; they also sought care more for LBP (61%) compared to the CM group (19%).
Harris	127	Systematic review	12 studies included; general population	CAM use in the US is growing and is being used by a greater proportion of the population.
Adams	128	Mail survey	42,000+; Australian women who consult alternative health practitioners	CAM users had poorer health status than non-CAM users, had lower levels of physical conditioning, and made more visits to GPs.

### Perceptions of CAM

#### Public Awareness

Emslie [133] studied changes in awareness of, use and attitudes toward CAM. This population survey demonstrated that use of CAM had increased during that period from 29% to 41%, with the greatest growth seen in reflexology. Fewer people had concerns about using CAM in 1999 (20%) compared to 1993 (25%). Fewer people were concerned about costs as well. The most common use of CAM was for headache and/or musculoskeletal pain, and most people found the CAM interventions effective. About one-third informed their GP that they had used CAM. Chiropractic had grown from 4% in 1993 to 9% in 1999, and from 56% knowing about chiropractic in 1993 to 70% in 1999.

#### Physician Attitudes

Lewith [134] looked at the attitudes and use of CAM amongst physicians in the United Kingdom. Over 12,000 physicians were surveyed; nearly 32% used some form of CAM themselves. Can was used more by those physicians in private practice compared to those in the National Health Service. Acupuncture and manipulative medicine (including both osteopathy and chiropractic) were the most commonly referred to practices. However, chiropractic was rarely used by practitioners (only about 0.6% used chiropractic care). Nonetheless, attitudes toward CAM in general were mainly positive. Ismail and Chan [135] provide information about primary care doctor perceptions of CAM in Perak, Malaysia. The question asked here were perceptions about harm; more than half of those surveyed felt that acupuncture, homeopathy and herbal medicines were potentially harmful, while 44% felt that manipulation could be harmful. Given this, nearly 60% used some form of CAM themselves and 67% had recommended to their patients that they seek CAM interventions. Nearly 9 in 10 were in favor of a hospital-based CAM center. Chan and Wong [136] looked at physician attitudes toward CAM in Hawaii, finding that chiropractic rated highly as having a role in conventional medicine, that many physicians would refer to chiropractors on behalf of their patients and that MS problems were reasons for seeking that care. And finally, there is one study [137] examining attitudes of first year medical students toward CAM. Of the 150 students in the survey, about 37% had used at least one form of CAM, with aromatherapy (51%) and homeopathy (30%) rating highest. In one of the more interesting findings in this study, students were asked to rate a series of CAM practices from their being skeptical about that discipline to being totally convinced about it. Chiropractic rated highest here (median score 8 on a scale of 10 with 1: Extremely Skeptical to 10: Convinced).

#### Military Veterans

one study [138] used a set of focus groups, totaling 100 veterans. The people in this study had criticisms of conventional medical care that centered around an over-reliance on the use of medications, and this was a direct cause of their willingness to seek CAM practitioners. They also noted that conventional medicine's lack of holism is also an important motivating factor for seeking CAM. They wish more involvement in their own care.

Table 7 presents summary results for the papers discussing perceptions toward CAM, and also includes information on design and response rates.

**Table 7:** Summary table of papers discussing perceptions toward CAM.

**Name**	**Ref**	**Design**	**N/Np**	**Main Results**
Emslie	132	Mail survey	800/432 (54%); USA	CAM use increased from 29% to 41% over the study period; concerns about costs and safety of using CAM had decreased; chiropractic use had grown from 4% to 9%, and awareness from 56% to 70% over the study period.
Lewith	133	Mail survey	12168/2875 (24%); Great Britain	MDs rarely used chiropractic care (0.6%), but attitudes toward CAM were generally positive.
Ismail	134	Mail survey	40/34 (85%); Kinta District, Perak	44% felt that manipulation could be harmful, but nearly 60% of physicians surveyed used some form of CAM and were in favor of a hospital-based CAM center.
Chan	135	Mail survey	1713/279 (16%); Hawaii-based physicians	Chiropractic rated highly as having a role in conventional medicine; many would refer patients to chiropractors.
Greenfield	136	Student questionnaire	150; first-year medical students	37% had used at least 1 form of CAM, with aromatherapy and homeopathy highest; chiropractic was seen as the most convincing form of CAM
Kroesen	137	Focus groups	100 people in 12 focus groups; US military veterans	People used CAM because they had negative feelings toward the over-prescription of medications. They also wanted more involvement in their own care.

## Conclusion

A review of the literature concerning chiropractic and CAM utilization breaks down into 7 categories: back pain papers, utilization papers, geographic population studies, access and insurance papers, papers examining CAM use in specific patient populations, CAM in specific settings and perceptions of CAM. Studies looking at chiropractic utilization demonstrate that the rates vary, but generally fall into a range from around 6% to 12% of the population [5,6,8,11], most of whom seek chiropractic care for low back pain and not for organic disease or visceral dysfunction [5,9,13-16,54,55,58]. CAM is itself used by people suffering from a variety of conditions [92-119], though it is often used not as a primary intervention, but rather as an additional form of care; the literature demonstrates that people usually do not let their primary medical physician know that they are using CAM [49,50]. CAM and chiropractic often offer lower costs for comparable results compared to conventional medicine [73,74,77,78,81,84,85]. It is apparent that the use of chiropractic is growing, though the impact remains modest; however, CAM as a whole is seeing wholesale increases in utilization.

This gives rise to many challenges. The health care environment, at least in the United States, is becoming more and more based on a combination of managed care and evidence-based medicine. This combination means that the chiropractic profession has to continue to provide the evidence that will allow the profession to obtain reimbursement for services. Certainly, there is good information that the addition of chiropractic services to medical care can reduce cost [73,84,85], but there is precious little information on specific interventions nor what kinds of patients respond best to what kinds of chiropractic care.

There is not enough clinical data for the management of various chiropractic problems, as noted by the findings here; however, chiropractors still treat patients who have conditions that are not related to the musculoskeletal system. The best evidence, both clinically and from the perspective of health services research, is for LBP. The findings here indicate that despite the literature that exists, there still are many gaps that need to be filled. There are, for example, few papers that do look at the impact that chiropractic care has in an integrated medical/chiropractic system. More needs to be done.

The paper presented here is a start to any effort that will examine the use of chiropractic and CAM for most conditions seen by the chiropractic profession, and it provides guidance as to what information exists. Future work will certainly help to elaborate the impact chiropractic has on health care worldwide.

## List of Abbreviations

AHCPR: Agency for Health Care Policy and Research

CAM: Complementary and Alternative Medicine

CD4: Cluster of Differential

CI: Confidence Interval

CINAHL: Cumulative Index to Nursing and Allied Health Literature

CISCOM: Centralized Information Service for Complementary Medicine

CM: Conventional Medicine

CPT: Current Procedural Terminology

CSAG: Clinical Standards Advisory Group

CT: Complementary Therapy

DC: Doctor of Chiropractic

DO: Doctor of Osteopathy

ECAM: Established Complementary and Alternative Medicine

ER: Emergency Room

GP: General Practitioner

HIE: Health Insurance Experiment

HIV: Human Immunodeficiency Virus

HMO: Health Maintenance Organization

HVLA: High-Velocity Low-Amplitude

ICD: International Classification of Disease

IPA: Independent Provider Association

LBP: Low Back Pain

MANTIS: Manual, Alternative and Natural Therapy Index System

MD: Medical Doctor

MEDSTAT: Medical Statistics Database

MS: Musculoskeletal

MS: Multiple Sclerosis

ND: Doctor of Naturopathy

NHS: National Health Service

NMS: Neuromusculoskeletal

ON: Ontario

OR: Odds Ratio

OTC: Over-the-Counter

PT: Physical Therapist

SD: Standard deviation

US: United States

USD United States Dollars

## Competing interests

The author(s) declare that they have no competing interests.

## Authors' contributions

DJL conducted the initial literature review and prepared the first draft of the manuscript. WM participated in the conception and design of the study and in the revision and coordination of the final manuscript. Both authors read and approved the final manuscript.

## References

[B1] Wolsko PM, Eisenberg DM, Davis RB, Kessler R, Phillips RS (2003). Patterns and perceptions of care for treatment of back and neck pain: results of a national survey. Spine.

[B2] Bigos S, Bowyer O, Braen G (1994). Acute low back problems in adults. Clinical practice guideline No. 14.

[B3] Shekelle PG (1994). The use and costs of chiropractic care in the health insurance experiment.

[B4] Eisenberg DM, Kessler RC, Foster C, Morlock FE, Calkins DR, Delbanco TL (1993). Unconventional medicine in the United States: prevalence, costs, and patterns of use. N Engl J Med.

[B5] Hurwitz EL, Coulter ID, Adams AH, Genovese BJ, Shekelle PG (1998). Use of chiropractic services from 1985 through 1991 in the Unites States and Canada. Am J Public Health.

[B6] Shekelle PG, Brook RH (1991). A community-based study of the use of chiropractic services. Am J Public Health.

[B7] von Kuster T (1980). Chiropractic health care: a national study of cost of education. Service utilization, number of practicing doctors of chiropractic and other key policy issues.

[B8] Cote P, Cassidy JD, Carroll L (2001). The treatment of neck and low back pain: Who seeks care? Who goes where?. Med Care.

[B9] Kelner M, Wellman B (1997). Who seeks alternative health care? A profile of the users of five models of care. J Alternative Compl Med.

[B10] Walker BF, Muller R, Grant WD (2004). Low back pain in Australian adults: health provider utilization and care seeking. J Manipulative Physiol Ther.

[B11] Sherman KJ, Cherkin DC, Connelly MT, Erro J, Savetsky JB, Davis RB, Eisenberg DM (2004). Complementary and alternative medical therapies for chronic low back pain: what treatments are patients willing to try?. BMC Complement Altern Med.

[B12] Caswell AM, West J (2002). An investigation into the factors affecting patient selection of chronic low back management methods, with particular emphasis to non-utilization of the complementary therapies, in the United Kingdom. J Back Musculoskeletal Rehabil.

[B13] Sundararajan V, Konrad TR, Garrett J, Carey T (1998). Patterns and determinants of multiple provider use in patients with acute low back pain. J Gen Intern Med.

[B14] Scheurmier N, Breen AC (1998). A pilot study of the purchase of manipulation services for acute low back pain in the United Kingdom. J Manipulative Physiol Ther.

[B15] Jamison JR (1995). Chiropractic referral: the views of a group of conventional medical practitioners with an interest in unconventional therapies. J Manipulative Physiol Ther.

[B16] Leboeuf-Yde C, Hennius B, Leufvenmark P, Thunman M (1997). Chiropractic in Sweden: a short description of patients and treatments. J Manipulative Physiol Ther.

[B17] Cherkin DC, Deyo RA, Sherman K, Erro JH, Hrbek A, Davis RB, Eisenberg DM (2002). Characteristics of visits to licensed acupuncturists, chiropractors, massage therapists, and naturopathic physicians. J Am Board Fam Prac.

[B18] (2000). 1998 National Ambulatory Medical Care Survey, CD-ROM series 13, no. 24.

[B19] Feuerstein M, Marcus SC, Huang GD (2004). National trends in non-operative care for nonspecific back pain. Spine J.

[B20] Weiner DK, Ernst E (2004). Complementary and alternative approaches to the treatment of persistent musculoskeletal pain. Clin J Pain.

[B21] Ernst E, Harkness EF (2001). Spinal manipulation: a systematic review of sham-controlled, double-blind, randomized clinical trials. J Pain Symptom Manage.

[B22] Druger M, Graves JE, Mayer JM, Miller J, Ploutz-Snyder LL, Uderman EE, Verna JL (2003). Exercise therapy for low back pain: chiropractors' patterns of use and perceptions of educational quality. J Chiropr Ed.

[B23] Whitman JA, Fritz JM, Childs JD (2004). The influence of experience and specialty certifications on clinical outcomes for patients with low back pain treated within a standardized physical therapy management program. J Orthop Sports Phys Ther.

[B24] Smith M, Stano M (1997). Costs and recurrences of chiropractic and medical episodes of low-back care. J Manipulative Physiol Ther.

[B25] Sun C, Desai GJ, Pucci DS, Jew S (2004). Musculoskeletal disorders: does the osteopathic medical profession demonstrate its unique and distinctive characteristics?. JAOA.

[B26] Simpson JK (1998). A study of referral patterns among Queensland general medical practitioners to chiropractors, osteopaths, physiotherapists and other. J Manipulative Physiol Ther.

[B27] Layton R (1986). Benefits Review Committee, second report. Chapter 10 – Chiropractic 1986.

[B28] McCann J, Phillips RL, Green LA, Fryer GE (2004). Chiropractors are not a usual source of primary health care. http://www.graham-center.org/onepager28.xml.

[B29] Sharma R, Haas M, Stano M (2003). Patient attitudes, insurance, and other determinants of self-referral to medical and chiropractic physicians. Am J Public Health.

[B30] Jain N, Astin JA (2001). Barriers to acceptance: an exploratory study of complementary/alternative medicine disuse. J Alt Compl Med.

[B31] Pirotta MV, Cohen MM, Kotsirilos V, Farish SJ (2000). Complementary therapies: have they become accepted in general practice?. Med J Australia.

[B32] Astin JA, Marie A, Pelletier KR, Hansen E, Haskell WL (1998). A review of the incorporation of complementary and alternative medicine by mainstream physicians. Arch Intern Med.

[B33] Ernst E, Resch KL, White AR (1995). Complementary medicine: what physicians think of it: meta-analysis. Arch Intern Med.

[B34] Goldszmidt M, Levitt C, Duarte-Franco E, Kaczorowski J (1995). Complementary health care services: a survey of general practitioners' views. CMAJ.

[B35] Verhoef MJ, Sutherland LR (1995). Alternative medicine and general practitioners: opinions and behaviors. Can Fam Phys.

[B36] Perkin MR, Pearcy RM, Fraser JS (1994). A comparison of the attitudes shown by general practitioners, hospital doctors and medical students towards alternative medicine. J R Soc Med.

[B37] Anderson E, Andersson P (1987). General practitioners and alternative medicine. J R Coll Gen Pract.

[B38] Wharton R, Lewith G (1986). Complementary medicine and the general practitioner. Br Med J.

[B39] Marshall RJ, Gee R, Israel M, Neave D, Edwards F, Dumble J, Wong S, Chan C, Patel R, Poon P (1990). The use of alternative therapies by Auckland general practitioners. NZ Med J.

[B40] Hadley CM (1988). Complementary medicine and the general practitioner: a survey of general practitioners in the Wellington area. NZ Med J.

[B41] Reilly DT (1983). Young doctors' views on alternative medicine. Br Med J.

[B42] Berman BM, Singh BK, Lao L, Singh BB, Ferentz KS, Hartnoll SM (1995). Physician attitudes toward complementary or alternative medicine: a regional survey. J Am Board Fam Pract.

[B43] Borkan J, Neher J, Ansen O, Smoker B (1994). Referrals for alternative therapies. J Fam Pract.

[B44] Cherkin D, MacCornack FA, Berg AO (1988). Managing of back pain: a comparison of the beliefs and behaviors of family physicians and chiropractors. West J Med.

[B45] Goldstein MS, Sutherland C, Jaffe DT, Wilson J (1988). Holistic physicians and family practitioners: similarities, difference and implications for health policy. Soc Sci Med.

[B46] Hawk C, Byrd L, Jansen RD, Long CR (1999). Use of complementary healthcare practices among chiropractors in the United States: a survey. Altern Ther Health Med.

[B47] Berman BM, Singh BK, Lao L, Singh BS, Ferentz KS, Hartjnoll SM (1995). Physicians' attitudes toward complementary or alternative medicine: a regional survey. J Am Board Fam Pract.

[B48] Smith M, Carber L (2002). Chiropractic health care in health professional shortage areas in the United States. Am J Public Health.

[B49] Eisenberg DM, Davis RB, Ettner SL, Appel S, Wilkey SA, Van Rompay MI, Kessler RC (1998). Trends in alternative medicine use in the United States, 1991–1997: results of a follow-up national survey. JAMA.

[B50] Eisenberg DM, Kessler RC, Van Rompay MI, Kaptchuk TJ, Wilkey SA, Appel S, Davis RB (2001). Perceptions among complementary therapies relative to conventional therapies among adults who use both: results from a national survey. Ann Intern Med.

[B51] Kessler RC, Davis RB, Foster DF, Van Rompay MI, Kaptchuk TJ, Wilkey SA, Appel S, Davis RB (2001). Long-term trends in the use of complementary and alternative medical therapies in the United States. Ann Intern Med.

[B52] Ernst E, White A (2000). The BBC survey of complementary medicine use in the UK. Compl Ther Med.

[B53] von Gruenigen VE, Showalter AL, Gil KM, Frasure HE, Hopkins MP, Jenison EL (2001). Complementary and alternative medicine use in the Amish. Compl Ther Med.

[B54] Yamashita H, Tsukayama H, Sugishita C (2002). Popularity of complementary and alternative medicine in Japan: a telephone survey. Compl Ther Med.

[B55] Barnes PA, Powell-Griner E, McFann K, Nahin R (2004). Complementary and alternative medicine use among adults: United States, 2002. Advance data from vital and health statistics, no. 343.

[B56] Factor-Litvak P, Cushman LF, Kronenberg F, Wade C, Kalmuss D (2001). Use of complementary and alternative medicine in New York City: a pilot study. J Alt Compl Med.

[B57] Smith M, Carber L (2002). A compilation of chiropractic and complementary/alternative medicine (CAM) data from public-use national surveys: report on a health-services research resource for the chiropractic and CAM scientific community. J Manipulative Physiol Ther.

[B58] Hawk C, Long C (1999). Factors affecting use of chiropractic services in seven Midwestern states of the United States. J Rural Health.

[B59] Dishman JD, Katz P (2003). Establishment of chiropractic services in a geriatric inpatient rehabilitation hospital: a pilot of one model of integration. J Chiropr Ed.

[B60] Nelson CD, McMillan DL, Richards DG, Mein EA, Redwood D (2000). Manual healing diversity and other challenges to chiropractic integration. J Manipulative Physiol Ther.

[B61] (1990). ACA Department of Statistics completes 1989 survey. J Manipulative Physiol Ther.

[B62] Nansel D, Szlazak M (1995). Somatic dysfunction and the phenomenon of visceral disease simulation: a probable explanation for the apparent effectiveness of somatic therapy in patients presumed to be suffering from true visceral disease. J Manipulative Physiol Ther.

[B63] Nelson CF, Lawrence DJ, Triano JJ, Bronfort G, Perle SM, Metz D, Hegetschweiler K, LaBrot T (2005). Chiropractic as spine care: a model for the profession. Chiropr Osteo.

[B64] Konrad TR, Fletcher GS, Carey TS (2004). Interprofessional collaboration and job satisfaction of chiropractic physicians. J Manipulative Physiol Ther.

[B65] Gensler WM (1990). The place of chiropractors in health care delivery: a case study of North Carolina. Soc Sci Med.

[B66] Coulehan JL (1985). Chiropractic and the clinical art. Soc Sci Med.

[B67] Anderson RT (1981). Medicine, chiropractic and caste. Anthrop Q.

[B68] McCorkle T (1961). Chiropractic: a deviant theory of disease and treatment in contemporary western culture. Hum Org.

[B69] Cleary PD (1982). Chiropractic use: a test of several hypotheses. Am J Public Health.

[B70] Mainous AG, Gill JM, Zoller JS, Wolman MG (2000). Fragmentation of patient care between chiropractors and family physicians. Arch Fam Med.

[B71] Cleary-Guida MB, Okvat HA, Oz MC, Ting W (2001). A regional survey of insurance coverage for complementary and alternative medicine: current status and future ramifications. J Alt Compl Med.

[B72] Metz RD, Nelson CF, LaBrot T, Pelletier KR (2004). Chiropractic care: is it substitution care or add-on care in corporate medical plans?. J Occup Environ Med.

[B73] Shekelle PG, Rogers WH, Newhouse JP (1996). The effect of cost sharing on the use of chiropractic services. Med Care.

[B74] Newhouse JP, the Insurance Experiment Group (1993). Free for all? Lessons from the RAND health insurance experiment.

[B75] Gordon NP, Lin TY (2004). Use of complementary and alternativemedicine by the adult membership of a large northern California health maintenance organization, 1999. J Ambulatory Care Manage.

[B76] Stano M (1993). A comparison of health care costs for chiropractic and medical patients. J Manipulative Physiol Ther.

[B77] Stano M, Smith M (1996). Chiropractic and medical costs of low back care. Med Care.

[B78] Lind BK, Lafferty WE, Tyree PT, Sherman KJ, Deyo RA, Cherkin DC (2005). The role of alternative medical providers for the outpatient treatment of insured patients with back pain. Spine.

[B79] Thomas KJ, Nicholl JP, Coleman P (2001). Use and expenditure on complementary medicine in England: a population based survey. Comp Ther Med.

[B80] Phelan SP, Armstrong RC, Knox DG, Hubka MJ, Ainbinder DA (2004). An evaluation of medical and chiropractic provider utilization and costs: treating injured workers in North Carolina. J Manipulative Physiol Ther.

[B81] Arcury TA, Preisser JS, Gesler WM, Sherman JE (2004). Complementary and alternative medicine use among rural residents in western North Carolina. Compl Health Practice Rev.

[B82] Gray CM, Tan AW, Pronk NP, O'Connor PJ Complementary and alternative medicine use among health plan members: a cross-sectional survey. Am College Phys.

[B83] Sarnat RL, Winterstein JF (2004). Clinical and cost outcomes of an integrative medicine IPA. J Manipulative Physiol Ther.

[B84] Legoretta AP, Metz RD, Nelson CF, Ray S, Chernicoff HO, DiNubile NA (2004). Comparative analysis of individuals with and without chiropractic coverage. Arch Intern Med.

[B85] Thomas KJ, Nicholl JP, Fall M (2001). Access to complementary medicine via general practice. Br J Gen Practice.

[B86] Hansen JP, Futch DB (1997). Chiropractic services in a staffmodel HMO: utilization and satisfaction. HMO Prac.

[B87] Stewart D, Weeks J, Bent S (2001). Utilization, patient satisfaction, and cost implications of acupuncture, massage and naturopathic medicine offered as covered health benefits: a comparison of two delivery models. Alt Ther Health Med.

[B88] Sawni-Sakand A, Schubiner H, Thomas RL (2002). Use of complementary/alternative therapies among children in primary care pediatrics. Ambulatory Ped.

[B89] Wilson KW, Klein JD (2002). Adolescents' use of complementary and alternative medicine. Ambulatory Ped.

[B90] Ramsey SD, Spencer AC, Topolski TD, Belza B, Patrick DL (2001). Use of alternative therapies by older adults with osteoarthritis. Arth Care Res.

[B91] VandeCreek L, Rogers E, Lester J (1999). Use of alternative therapies among breast cancer outpatients compared with the general population. Alt Ther Health Med.

[B92] Shen J, Anderson R, Albert PS, Wengler N, Glaspy J, Cole M, Shekelle P (2002). Use of complementary/alternative therapies by women with advanced-stage breast cancer. BMC Compl Alt Med.

[B93] Shinto L, Calabrese C, Morris C, Sinsheimer S, Bourdette D (2004). Complementary and alternative medicine in multiple sclerosis: survey of licensed naturopaths. J Alt Compl Med.

[B94] Nayak S, Matheis RJ, Schoenberger NE, Shiflett SC (2003). Use of unconventional therapies by individuals with multiple sclerosis. Clin Rehabil.

[B95] Stude DE, Mick T (1993). Clinical presentation of a patient with multiple sclerosis and response to manual chiropractic adjustive therapies. J Manipulative Physiol Ther.

[B96] Furler MD, Einarson TR, Wlamsley S, Millson M, Bendayan R (2003). Use of complementary and alternative medicine by HIV-infected outpatients in Ontario, Canada. AIDS Patient Care STD.

[B97] Bica I, Tang AM, Skinner S, Spiegelman D, Knox T, Gorbach S, Wilson IB (2003). Use of complementary and alternative therapies by patients with human immunodeficiency virus disease in the era of highly active antiretroviral therapy. J Alt Compl Med.

[B98] Wiwanitkit W (2003). The use of CAM by HIV-positive patients in Thailand. Compl Ther Med.

[B99] Bronfort G, Evans R, Kubic P, Filkin P (2001). Chronic pediatric asthma and chiropractic spinal manipulation: a prospective clinical series and randomized clinical pilot study. J Manipulative Physiol Ther.

[B100] Balon J, Aker PD, Crowther ER, Danielson C, Cox PG, O'Shaughnessy D, Walker C, Goldsmith CH, Duku E, Sears MR (1998). A comparison of active and simulated chiropractic manipulation as adjunctive treatment for childhood asthma. N Engl J Med.

[B101] Blanc PD, Trupin L, Earnest G, Katz PP, Yelin EH, Eisner MD (2001). Alternative therapies among adults with a reported diagnosis of asthma or rhinosinusitis: data from a population-based survey. Chest.

[B102] Ernst E, Cassileth BR (1998). The prevalence of complementary/alternative medicine in cancer: a systematic review. Cancer.

[B103] Lewith GT, Broomfield J, Prescott P (2002). Complementary cancer care in Southampton: a survey of staff and patients. Compl Med Ther.

[B104] Rees R, Feigel I, Vickers A, Zollman C, McGurk R, Smith C, Eade S (1999). Use of complementary therapies by women with breast cancer in the South Thames Region. A short report of a research report funded by the NHS Executive South Thames. The Research Council for Complementary Medicine (Report).

[B105] Ceylan S, Hamzaoglu O, Komurcu S, Beyan C, Yalcin A (2002). Survey of the use of complementary and alternative medicine among Turkish cancer patients. Compl Ther Med.

[B106] Harris P, Finlay IG, Cook A, Thomas KJ, Hood K (2003). Complementary and alternative medicine use by patients with cancer in Wales: a cross sectional survey. Compl Ther Med.

[B107] Unutzer J, Klap R, Sturm R, Young AS, Marmon T, Shatkin J, Wells KB (2000). Mental disorders and the use of alternative medicine: results from a national survey. Am J Psych.

[B108] Kessler RC, Soukup J, Davis RB, Foster DF, Wilkey SA, Van Rompay MI, Eisenberg D (2001). The use of complementary and alternative therapies to treat anxiety and depression in the United States. Am J Psych.

[B109] Simon G, Cherkin DC, Sherman KJ, Eisenberg DM, Deyo RA, Davis RB (2004). Mental health visits to complementary and alternative providers. Gen Hosp Psych.

[B110] Demling JH, Neubauer S, Luderer HJ, Worthmuller M (2002). A survey on psychiatric patients' use fo non-medical alternative practitioners: incidence, methods, estimation, and satisfaction. Compl Ther Med.

[B111] McPherson M, Arango P, Fox H, Lauver C, McManus M, Newacheck PW, Perrin JM, Shonkoff JP, Strickland B (1998). A new definition of children with special health care needs. Pediatrics.

[B112] Sander H, Davis MF, Duncan B, Meaney FJ, Haynes J, Barton LL (2003). Use of complementary and alternative medical therapies among children with special health care needs in Southern Arizona. Pediatrics.

[B113] Schoenberg NE, Stoller EP, Kart CS, Perzynski A, Chapleski EE (2004). Complementary and alternative medicine use among a multiethnic sample of older adults with diabetes. J Alt Compl Med.

[B114] Rolniak S, Browning L, MacLeod BA, Cockley P (2004). Complementary and alternative medicine use among urban ED patients: prevalence and patterns. J Emerg Nurs.

[B115] Li JZ, Quinn JV, McCullough CE, Jacobs BP, Chan PV (2004). Patterns of complementary and alternative medicine use in ED patients and its association with health care utilization. Am J Emerg Med.

[B116] Brunelli B, Gorson KC (2004). The use of complementary and alternative medicines by patients with peripheral neuropathy. J Neurol Sci.

[B117] Wang SM, Caldwell-Andrews AA, Kain ZN (2003). The use of complementary and alternative medicines by surgical patients: a follow-up survey study. Anesth Analg.

[B118] Kitai E, Vinker S, Sandiuk A, Hornik O, Zeltcer C, Gaver A (1998). Use of complementary and alternative medicine among primary care patients. Fam Pract.

[B119] Cuellar N, Aycock T, Cahill B, Ford J (2003). Complementary and alternative (CAM) use by African American (AA) and Caucasian American (CA) older adults in a rural setting: a descriptive, comparative study. BMC Complement Altern Med.

[B120] Foster DF, Phillips RF, Hamel B, Eisenberg D (2000). Alternative medicine use in older Americans. J Am Geriatr Soc.

[B121] Polipnick J, Hondras M, Delavan S, Lawrence DJ (2005). An exploration of community leader perspectives about minority involvement in chiropractic research. J Alt Compl Med.

[B122] Keegan L (1996). Use of alternative therapies among Mexican Americans in the Texas Rio Grande Valley. J Holisitic Nurs.

[B123] Sirois FM (2002). Treatment seeking and experience with complementary/alternative medicine: a continuum of choice. J Alt Compl Med.

[B124] Astin JA (1998). Why patients use alternative medicine. Results of a national study. JAMA.

[B125] Ramos-Remus C, Watters CA, Dyke L, Suarez-Almazor ME (1999). Assessment of health locus of control in the use of non-conventional remedies by patients with rheumatic disease. J Rheumatol.

[B126] Blais R, Maiga A, Aboubacar A (1997). How different are users and non-users of alternative medicine?. Can J Public Health.

[B127] Harris P, Rees R (2000). The prevalence of complementary and alternative medicine use among the general population: a systematic review of the literature. Compl Med Ther.

[B128] Centralized Information Service for Complementary Medicine. http://www.rccm.org.uk/ciscom/CISCOM_intro.aspx.

[B129] Adams J, Sibbritt DW, Easthope G, Young AF (2003). The profile of women who consult alternative health practitioners in Australia. Med J Australia.

[B130] Brown WJ, Bryson L, Byles JE, Dobson AJ, Lee C, Mishra G, Schonfield M (1998). Women's Health Australia: recruitment for a national longitudinal cohort study. Women Health.

[B131] Giordano J, Garcia MK, Boatwright D, Klein K (2003). Complementary and alternative medicine in mainstream public health: a role for research in fostering integration. J Alt Compl Med.

[B132] O'Brien K (2004). Complementary and alternative medicine: the move into mainstream health care. Clin Exp Optom.

[B133] Emslie MJ, Campbell MK, Walker KA (2002). Changes in public awareness of, attitudes to, and use of complementary therapy in North East Scotland: surveys in 1993 and 1999. Compl Ther Med.

[B134] Lewith GT, Hyland M, Gray SF (2001). Attitudes to and use of complementary medicine among physicians in the United Kingdom. Compl Ther Med.

[B135] Ismail IA, Chan SC (2004). Knowledge and practice of complementary medicine amongst public primary care clinic doctors in Kinta District, Perak. Med J Malaysia.

[B136] Chan PS, Wong MM (2004). Physicians and complementary-alternative medicine: training, attitudes, and practices in Hawaii. Hawaii Med J.

[B137] Greenfield SM, Innes MA, Allan TF, Wearn AM (2002). First year medical students' perceptions and use of complementary and alternative medicine. Compl Ther Med.

[B138] Kroesen K, Baldwin CM, Brooks AJ, Bell IR (2002). US military veterans' perceptions of the conventional medical care system and their use of complementary and alternative medicine. Fam Pract.

